# An Acidic Loop and Cognate Phosphorylation Sites Define a Molecular Switch That Modulates Ubiquitin Charging Activity in Cdc34-Like Enzymes

**DOI:** 10.1371/journal.pcbi.1002056

**Published:** 2011-05-26

**Authors:** Elena Papaleo, Valeria Ranzani, Farida Tripodi, Alessandro Vitriolo, Claudia Cirulli, Piercarlo Fantucci, Lilia Alberghina, Marco Vanoni, Luca De Gioia, Paola Coccetti

**Affiliations:** Department of Biotechnology and Biosciences, University of Milano-Bicocca, Milan, Italy; MRC Laboratory of Molecular Biology, United Kingdom

## Abstract

E2 ubiquitin-conjugating enzymes are crucial mediators of protein ubiquitination, which strongly influence the ultimate fate of the target substrates. Recently, it has been shown that the activity of several enzymes of the ubiquitination pathway is finely tuned by phosphorylation, an ubiquitous mechanism for cellular regulation, which modulates protein conformation. In this contribution, we provide the first rationale, at the molecular level, of the regulatory mechanism mediated by casein kinase 2 (CK2) phosphorylation of E2 Cdc34-like enzymes. In particular, we identify two co-evolving signature elements in one of the larger families of E2 enzymes: an acidic insertion in β4α2 loop in the proximity of the catalytic cysteine and two conserved key serine residues within the catalytic domain, which are phosphorylated by CK2. Our investigations, using yeast Cdc34 as a model, through 2.5 µs molecular dynamics simulations and biochemical assays, define these two elements as an important phosphorylation-controlled switch that modulates opening and closing of the catalytic cleft. The mechanism relies on electrostatic repulsions between a conserved serine phosphorylated by CK2 and the acidic residues of the β4α2 loop, promoting E2 ubiquitin charging activity. Our investigation identifies a new and unexpected pivotal role for the acidic loop, providing the first evidence that this loop is crucial not only for downstream events related to ubiquitin chain assembly, but is also mandatory for the modulation of an upstream crucial step of the ubiquitin pathway: the ubiquitin charging in the E2 catalytic cleft.

## Author Summary

A major mechanism for promoting protein regulation in eukaryotes involves the labeling with ubiquitin molecules of target proteins. Protein ubiquitination is involved in almost all aspects of eukaryotic cellular functions and is mediated, at the molecular level, by a hierarchical cascade of three different enzymes. Among these enzymes, E2 ubiquitin-conjugating enzymes are located at the heart of the ubiquitination pathway and are key mediators of protein ubiquitination, which strongly influence the ultimate fate of the target substrates. Since several E2s have also been related to a variety of cancer and neurodegenerative disorders, increasing efforts are being devoted to the understanding of E2 regulation at the molecular level, a mandatory step for a complete understanding of the ubiquitination process. In the present contribution, we propose, by computational and biochemical investigations, a conserved mechanism of regulation by phosphorylation of the catalytic activity of a class of E2 enzymes, which plays a major role in the regulation of cell cycle progression and tumor development. Our results shed new light on and clarify molecular aspects related to one of the first steps of the ubiquitination cascade and its regulation.

## Introduction

A major mechanism for the promotion of protein regulation in eukaryotes involves the covalent attachment of ubiquitin (Ub), mediated by a hierarchical cascade of E1-E2-E3 enzymes [1], [2]. In particular, the E1 enzyme activates the ubiquitin in an ATP-dependent reaction and engages one of many cognate E2 ubiquitin-conjugating enzymes to initiate downstream events. Ubiquitin is covalently attached to the target protein through an isopeptide bond between the glycine in position 76 of ubiquitin and the ε-amino group of an internal lysine residue of the target protein, through the coordinate function of E3 ubiquitin-ligases. Several E2 cycles of E1-mediated ubiquitin loading/unloading lead to different polyubiquitination or monoubiquitination of the substrates. By multiple runs of reactions, ubiquitin is covalently attached to substrates to form K48-linked or K11-linked polyubiquitinated conjugates that are addressed to proteasomal degradation [3], [4], [5], [6], [7], [8]. Moreover, post-translational modification by Ub on other lysine residues, as K63, or ubiquitin-like proteins regulates several other processes, including cell division, immune responses and embryonic development [7], [9], [10], [11].

**Figure 1:**
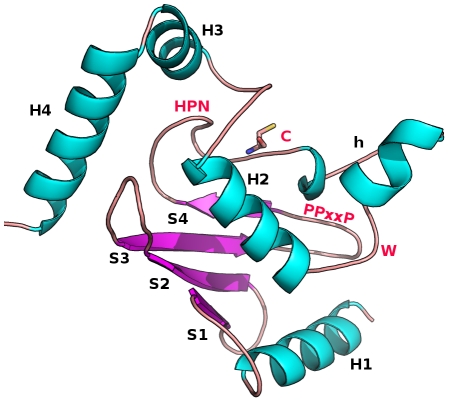
Summary structure of 3D UBC domain architecture. The 3D architecture of UBC domain of the E2 superfamily, as well as secondary structure elements and the conserved residues are shown. α-helices are labeled H1 to H4 (with h for a 3.10 helix common to some E2 structures) and β-strands are labeled S1 to S4. The catalytic cysteine (C), a strictly conserved tryptophan residue (W) and the HPN (His-Pro-Asn) motif in the proximity of the catalytic site, as well as the proline rich motif (PPxxP) are indicated [14]. The structure is adapted from the 3D structure of Ube2g2 (PDB code 2CYX).

The yeast and human genomes encode for tens of E2s, allowing for a multitude of distinct events [12], [13]. E2s have been classified in 17 families, by a comprehensive phylogenetic analysis of several complete genomes [14] and have been shown to present different electrostatic potential surface properties which could be related to their specificity [15], [16]. All E2 enzymes share a conserved catalytic core domain (Ub-conjugating domain, UBC) which in many cases is the minimal sufficient unit for their activity, containing the catalytic cysteine (Figure 1) and the interaction interfaces for E1 and E3 enzymes. The 150-residues UBC domain adopts a β/α fold (Figure 1) and the highly conserved catalytic cysteine is located in a shallow cleft interacting with the Ub C-terminal tail [17]. Despite conservation of the UBC domain fold, many E2s contain sequence insertions. Yeast and mammalian Cdc34 are multi-domain E2 enzymes, which catalyze the formation of polyubiquitin chains on several proteins involved in cell-cycle regulation [18]. Yeast Cdc34 is characterized by a 12-residue insertion (residues 103–114) in the β4α2 loop of the UBC domain, which is known as the “acidic loop” [19], [20]. Interestingly, the acidic insertion also occurs in other yeast and human homologs and forms an extended and flexible loop [21]. In fact, alanine-mutations of the β4α2 acidic residues abolish polyubiquitin chain assembly in the human E2 Ube2g2 [22]. In Cdc34, mutations of the acidic residues compromise the processivity and linkage specificity of polyubiquitin chain synthesis [22], [23], [24]. However, few details are still known about the structural and functional properties of the acidic insertion. Several E2 catalytic domains have been reported to be finely regulated by post-translational phosphorylations [25], [26], [27], [28]. Cdc34 is a substrate of casein kinase 2 (CK2) [25], [26], [29], [30], [31], a highly conserved kinase essential in several different cellular processes [32]. In particular, CK2 phosphorylates yeast Cdc34 within the C-terminal [26], [31] and the catalytic domain (S130 and S167) [25]. S130 and S167 phosphorylations are required to stimulate yeast Cdc34 ubiquitin-charging activity *in vitro* and to complement a *cdc34-2* ^ts^ mutant *in vivo* [25].

In light of the above scenario, we provide the first molecular model of the regulatory mechanism mediated by CK2 phosphorylation of E2 Cdc34-like catalytic domain. In particular, we show that the acidic loop conformation is modulated by phosphorylation of two key serine residues. Our investigation strongly suggests that this regulatory mechanism is a conserved feature of Cdc34-like E2s, a crucial element for the modulation of ubiquitin charging activity.

## Results

### Co-evolution of phosphorylation sites and acidic loop in Cdc34-like E2 enzymes

**Figure 2:**
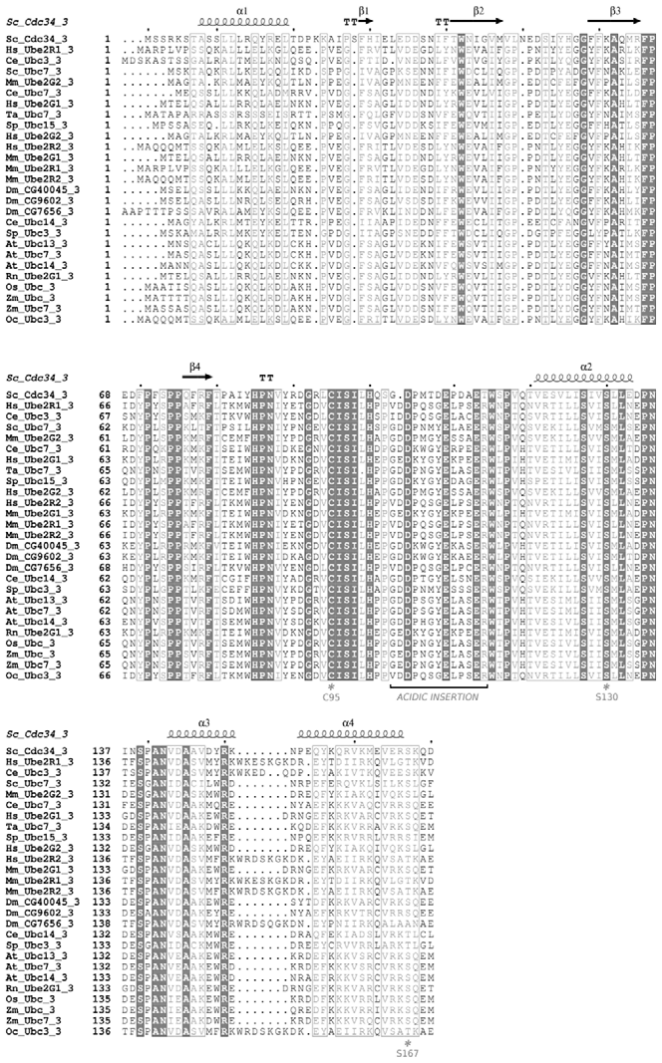
Multiple sequence alignment of family 3 E2s. Identical residues (grey-filled boxes) and similar residues (grey boxes) are indicated.

**Figure 3:**
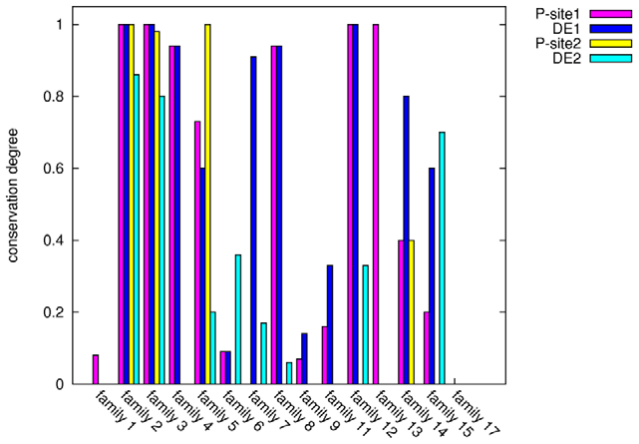
Conservation of phospho-sites and of CK2 consensus pattern. The conservation degree of S/T residues at positions corresponding to Cdc34 S130 (Psite-1) and S167 (Psite-2), as well as of acidic residues of the CK2 consensus pattern (DE1 and DE2) has been calculated from the intra-family sequence alignments in Figure 4S.

CK2 phosphorylation sites at S130 and S167 are functionally relevant for the ubiquitin-charging activity of yeast Cdc34 [25]. Multiple sequence and structural alignments among representative members of the different E2 families (Figure 2, Figure 1 in Text S1) show that phospho-sites corresponding to S130 and S167 of Cdc34, as well as the typical acidic consensus pattern for CK2 kinase, are strongly conserved in E2s belonging to family 3. E2 family 3 is the only family whose members show a 12/13 aminoacid insertion in the β4α2 loop (Figure 2, Figure 1 in Text S1). In the other E2 families, which lack the acidic insertion, the phospho-sites are poorly conserved, with the exception of E2 families (2, 4, 8, 12). The latter include members which are the closest homologs (percentage of identity >30%) to family 3 members and which are thus characterized by a higher degree of serine conservation (Figure 3). Interestingly, in most of the other E2 families, which lack the acidic insertion, serines are often replaced by non-phosphorylatable residues with no specific physical-chemical properties (Figure 2, Figure 1 in Text S1). These data suggest co-evolution of the phospho-sites at Cdc34 130 and 167 positions and the acidic loop in the proximity of the catalytic site in Cdc34-like E2 enzymes.

### Conformational changes of β4α2 loop modulates accessibility of the Cdc34^UBC^ catalytic cleft

The striking co-presence of phosphorylation sites and the acidic loop only in family 3 E2 enzymes, prompted us to test the hypothesis of an interconnected structural, functional and regulatory role of these two elements. First, the dynamic properties of Cdc34 and its homologs have been investigated by multiple molecular dynamics (MD) simulations of the non-phosphorylated UBC domains (Table 1). In particular, flexibility profiles, principal component analysis (PCA) and correlation between accessibility of the catalytic cleft and other regions of the protein structure have been calculated. In this context, PCA applied to MD simulations allows the identification of the most relevant displacements of groups of residues and emphasizes the amplitude and direction of the dominant protein motions, by projecting them on a subset of principal components (eigenvectors) of the Cα atoms covariance matrix calculated from the MD conformational ensemble [33].

**Table 1:** Summary of MD simulations and simulated systems.

Cdc34 variant	Number of replicas	Duration per replica	Total duration	Starting structure
*Cdc34^UBC^	12	20 ns	0.24 µs	Cdc34 models using *C.elegans* Ubc7 (replicas 1–4), Ube2g (replicas 5–8) and *S. cerevisiae* Ubc7 (replicas 9–12) as templates
Cdc34^UBC^-pS130-pS167	11	50 ns	0.55 µs	Average structure from ensembles A (replicas 1–3), B (replicas 4–5) and C (replicas 6–7), D (replicas 8–9), and E (replicas 10–11)
Cdc34^UBC^-pS130	10	50 ns	0.50 µs	Average structure from ensembles A (replicas 1–2), B (replicas 3–4) and C (replicas 5–6), D (replicas 7–8), and E (replicas 9–10)
Cdc34^UBC^-S130A-S167A	4	50 ns	0.20 µs	Average structure from ensemble A
Cdc34^UBC^-pS130-S167A	4	50 ns	0.20 µs	Average structure from ensemble A
Cdc34^UBC^-S130D	4	50 ns	0.20 µs	Average structure from ensembles A (replicas 1–2) and D (replicas 3–4)
Ubc7	4	40 ns	0.16 µs	X-ray structure of *S.cerevisiae* Ubc7 (pdb entry 2UCZ)
Ube2g2	4	40 ns	0.16 µs	X-ray structure of human Ube2g2 (pdb entry 2CYX or 1KLY)
Cdc34Δ12^UBC^	1	100 ns	0.1 µs	Average structure from ensemble A
Ubc1	1	40 ns	0.04 µs	X-ray structure of the catalytic domain of *S.cerevisiae* Ubc1 (pdb entry 1FYZ)

*In order to evaluate stability of the Cdc34^UBC^ replicas, sample simulations using as starting structure the Cdc34 model which has been generated using as template Ube2g2 NMR structure have been extended to 80 and 100 ns.

**Figure 4:**
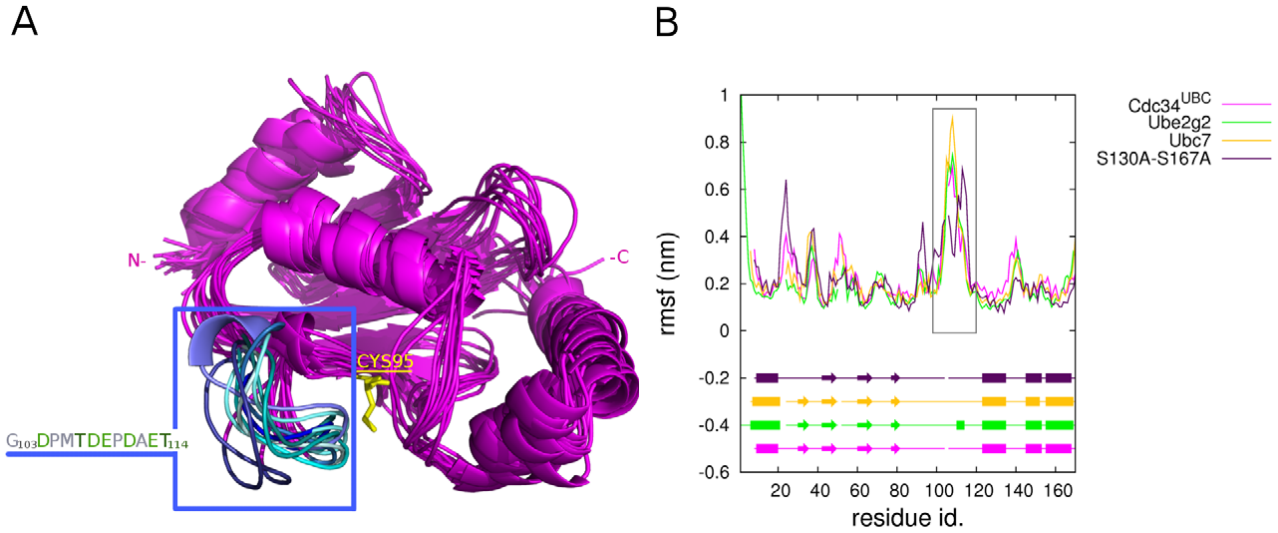
Cdc34^UBC^ simulations. (A) Average structures from Cdc34^UBC^ simulations. The acidic loop is shown in blue and its amino acid sequence is indicated in the box on the left. Acidic and polar residues of the acidic loop are shown in green. (B) Cα rmsf profiles computed on Ubc7, Ube2g2 and Cdc34^UBC^-S130AS167A macro-trajectories in comparison to Cdc34^UBC^. The most persistent secondary structures during the simulations are represented for each system. The rectangular box indicates the acidic loop.

The conformational sub-states distribution in the proximity of the native-state of Cdc34^UBC^ has been evaluated by free energy landscape (FEL) representation using as two-dimensional (2D) coordinates the first two principal components (PCs) derived by PCA (Figure 2 in Text S1), as well as from structural cluster analysis. The structural cluster analysis and the FEL representation indicate that Cdc34^UBC^ exists in several sub-states in solution (Figure 2 in Text S1, Figure 4A). In particular, it is possible to identify five main structural ensembles (labeled A–E) which correspond to the minimum free energy basins and the most populated structural clusters from cluster analysis. These structural ensembles capture the most relevant conformational and dynamical features of Cdc34^UBC^. The major differences among the ensembles are related to different conformations of the β4α2 loop (Figure 4A).

Interestingly, the β4α2 loop close to the E2 active site, and in particular its acidic insertion (residues 103–114) is characterized by a high conformational variability (Figure 4A), that can be described quantitatively by flexibility indexes as root mean square fluctuation (rmsf) calculated on each Cdc34^UBC^ ensemble trajectory (Figure 4B, Figure 3 in Text S1). MD simulations of Cdc34 homologs with known 3D structure (yeast Ubc7 and human Ube2g2) (Figure 4B) confirmed that the β4α2 loop features the highest conformational freedom also in other family 3 members. These results are in good agreement with evidence from X-ray crystallographic investigations of these enzymes [34], [35], in which the regions corresponding to the acidic loop seem to be disordered and associated to high B-factor values. Moreover, recent NMR studies of free Ube2g2 [21] or Ube2g2 in complex with a domain of its E3 partner [36] suggest the possibility of conformational changes of the β4α2 loop. However, the MD simulations carried out in the present work, allow a higher sampling of the conformation accessible to the β4α2 loop, highlighting larger structural rearrangements.

**Figure 5:**
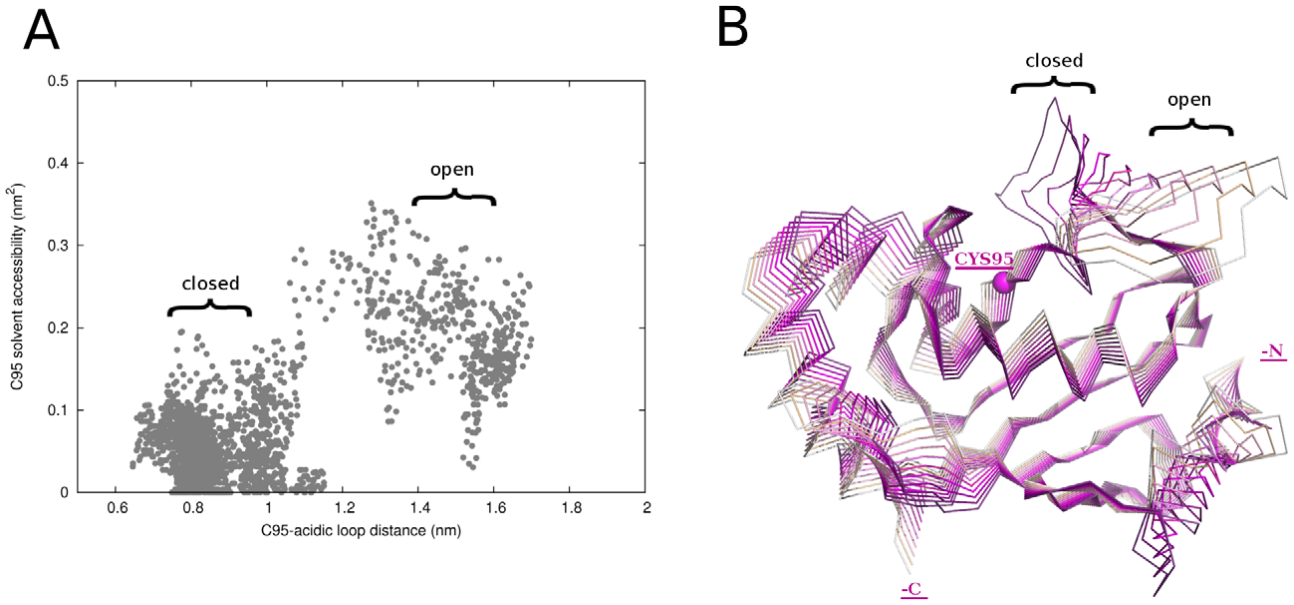
Conformational variability of the acidic loop and C95 solvent accessibility. (A) Distance between the centers of mass of C95 and the acidic loop as a function of solvent accessible surface (SAS) of C95 sidechain. (B) Projections of the simulations frames along the PC1 of Cdc34^UBC^ concatenated trajectory, indicated with different shades of magenta. The open and closed conformations of the acidic loop are indicated.

Figure 5A shows solvent accessibility of the catalytic C95 side chain as a function of the distances between the C95 and the acidic insertion. Solvent accessibility increases as the acidic loop “moves away” from the active site. We refer to conformations of the β4α2 loop in proximity of - and away from - the catalytic cysteine as “closed” and “open” conformations, respectively. Since PCA makes it possible to identify amplitude and direction of relevant protein motions, the projections of the first principal component (PC1) on the 3D structure of Cdc34^UBC^ have been analyzed. Protein motions governing PC1 are related to rearrangements and oscillations of the acidic loop over the catalytic site (Figure 5B) that make it function as a “lid” modulating the accessibility of the Ub-binding cleft.

### Phosphorylation of Cdc34^UBC^ improves accessibility of the catalytic site by displacing the acidic loop

**Figure 6:**
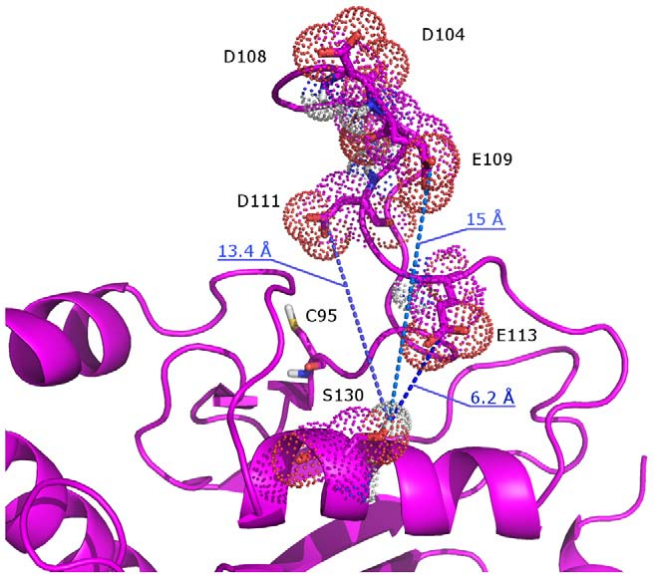
Mechanism of acidic loop activation by electrostatic repulsive effects. An average structure from Cdc34^UBC^ simulations is reported in which acidic residues (D104, D108, E109, D111 and E113) of the acidic loop, as well as S130 and catalytic C95, are shown. Acidic residues of the acidic loop come transiently close to the α-helix in which S130 is located.

**Figure 7:**
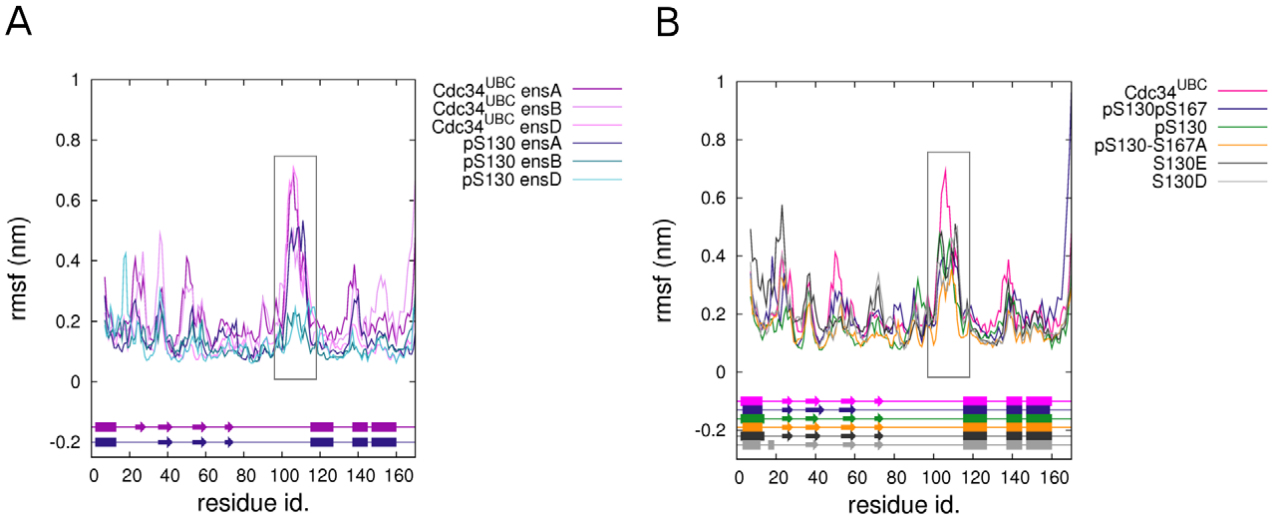
Flexibility profiles of phospho-Cdc34^UBC^ variants. (A) Cα rmsf of the ensemble trajectories of Cdc34^UBC^-pS130 and Cdc34^UBC^. (B) Cα rmsf of ensemble trajectories of Cdc34^UBC^-pS130, Cdc34^UBC^-pS130-S167A, Cdc34^UBC^-S130D, Cdc34^UBC^-S130E and Cdc34^UBC^-pS130pS167. The most persistent secondary structures during the simulations are represented schematically for each system. The rectangular box indicates the acidic loop.

In Cdc34^UBC^ simulations, some residues within the acidic insert (D104, D108, E109, D111 and E113), came transiently close to the catalytic cleft (Figure 5A, Figure 6) and, therefore also to the α-helix in which S130 is located. This observation suggests that S130 phosphorylation, introducing a strongly negatively charged group, may result in electrostatic repulsion with the above-mentioned acidic residues, favoring the open conformation of the β4α2 loop. This hypothesis was tested by MD simulations of phospho-variants of the Cdc34^UBC^ (Table 1) that were compared with simulations of the non-phosphorylated Cdc34^UBC^, as well as with simulations of the Cdc34^UBC^ alanine mutants, in which the phosphorylation sites (S130 and S167) are abolished, and that have been used in biochemical and genetic experiments [25]. While alanine substitutions do not influence secondary structure content and flexibility patterns described for native Cdc34^UBC^ (Figure 4B), flexibility profiles of Cdc34^UBC^-pS130 indicate that phosphorylation strongly reduces atomic fluctuations, structurally stabilizing the whole 3D architecture and in particular the acidic insertion (103–114) (Figure 7A). Phosphorylation of both S130 and S167 (Figure 4 in Text S1, Figure 7B) has comparable effects on acidic loop conformations compared to those observed for phosphorylated Cdc34^UBC^-pS130 (Figure 7), with a slightly higher capability of reducing protein flexibility of β4α2 loop of the double-phospho variant with respect to Cdc34^UBC^-pS130.

**Figure 8:**
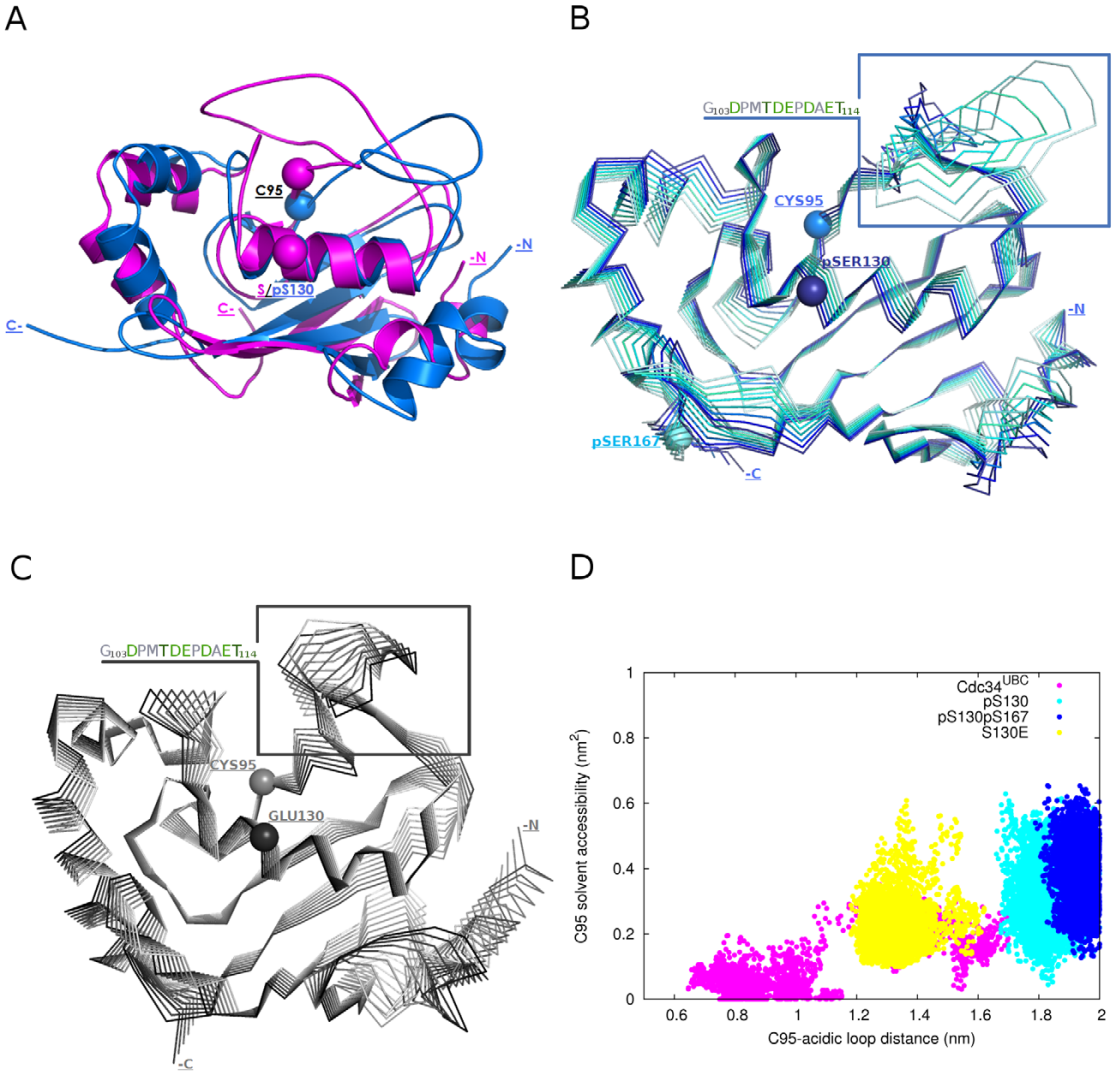
Phospho-Cdc34^UBC^ simulations. (A) Average structure from Cdc34^UBC^-pS130 (blue) simulations in comparison to a Cdc34^UBC^ (magenta) reference structure with the loop in a closed conformation. (B) Projection of the simulations frames along the PC1 of Cdc34^UBC^-pS130 concatenated trajectory, indicated with different shades of blue. (C) Projections of the simulations frames along the PC1 of Cdc34^UBC^-S130E concatenated trajectory indicated with different shades of grey. (D) Solvent accessible surface (SAS) of C95 sidechain in comparison to the distance between the centers of mass of C95 and the acidic loop in the Cdc34^UBC^, Cdc34^UBC^-pS130, Cdc34^UBC^-pS130-pS167 and Cdc34^UBC^-S130E simulations.

The comparison between snapshots of Cdc34^UBC^ and phospho-Cdc34^UBC^ simulations revealed that, in the phospho-Cdc34^UBC^ versions, the loop is stabilized in an open conformation (Figure 8A, in blue), determining a higher solvent accessibility of the catalytic residue C95. It is worth mentioning that phospho-Cdc34 structures have an average value of C95 solvent accessibility (avSAS = 30%) comparable to the average value calculated for the 40 ns simulation of yeast Ubc1 (avSAS = 34%), a family 2 E2 lacking the acidic insertion. Interestingly, solvent accessibility values of the catalytic cysteine side-chain in native Ube2g2 NMR structure [21], as well as in Ube2g2 in complex with a domain of its E3 partner [36] are lower than 15%, indicating that phosphorylation in the Cdc34 catalytic domain promotes a more relevant outward conformational displacement of the acidic loop than previously shown (Figure 5 in Text S1).

These data are confirmed by the analysis of the motions described by the PC1 of phospho-Cdc34^UBC^ simulations (Figure 8B). Fluctuations along PC1 are strongly reduced and the PCA analysis failed to identify the opening/closing oscillations of the acidic loop typical of Cdc34^UBC^, whereas a displacement of the loop toward open conformations is evident (Figure 5, 8B and D). In order to assess that effects induced by S130 phoshorylation are mainly due to electrostatic repulsion between acidic residues in the loop and the negatively charged pS130, simulations of mutant Cdc34^UBC^ variants with S130 replaced by negatively charged residues (Cdc34^UBC^-S130D and Cdc34^UBC^-S130E) have been carried out. The dynamical and structural properties of Cdc34^UBC^-S130D and Cdc34^UBC^-S130E, as well as the stabilization of the loop in an open conformation, are in agreement with data derived from phospho-Cdc34^UBC^ simulations (Figure 7B, 8C and Figure 6 in Text S1).

To quantitatively define differences in the solvent accessibility of catalytic cysteine and displacement of the acidic loop from the catalytic site, the distributions of the solvent accessibility of the catalytic C95 side chain as a function of the distances between C95 and the acidic insertion have been compared in non-phosphorylated, phospho- and S130E Cdc34^UBC^ variants (Figure 8D). Interestingly, the distribution of C95 SAS as a function of the distance between C95 and the acidic loop (Figure 8D), in agreement with analysis of rmsf profiles (Figure 7B), highlights a slightly greater displacement toward open conformations induced by the double-phospho variants. Moreover, the analysis suggests that the mutation of serine to acidic (for example glutamate) residues has lower effects in promoting acidic loop displacement than do the phospho-variants, which is probably related to enhanced electrostatic repulsion induced by the replacement of a poly-anionic phospho-serine with a mono-anionic residue (as Asp or Glu).

Our results suggest that the activation of Ub-conjugation activity by S130 phosphorylation is triggered by electrostatic repulsive effects between the phospho-Ser and the acidic residues of the β4α2 loop, promoting an outward displacement of the loop and a competent conformation of the catalytic cleft for Ub-charging.

### *In vitro* activation of Ub-charging activity of Cdc34 by CK2 phosphorylation requires the acidic loop

**Figure 9:**
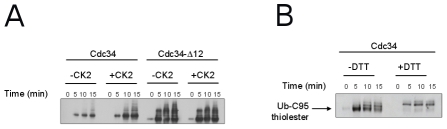
CK2 phosphorylation does not stimulate Cdc34-ubiquitin charging activity of a His_6_-Cdc34-Δ12 protein. (A) Recombinant His_6_-Cdc34 and His_6_-Cdc34-Δ12, either phosphorylated *in vitro* by CK2 or not, were incubated with E1, ATP and biotinylated ubiquitin. Samples were taken at the indicated time points. Data are representative of one of three experiments performed. (B) Recombinant His_6_-Cdc34 was treated as in (A) except that DTT (200 mM) was added to protein samples prior to electrophoresis.

According to the above-described model, phosphorylation by CK2 of the catalytic domain of Cdc34 can induce an outward displacement of the acidic loop, promoting Ub access to the catalytic cleft. Thus, to experimentally test the model, we investigated the effect of CK2 phosphorylation on the efficiency of Ub-charging of a protein lacking the 12 residue insertion in the acidic loop (His_6_-Cdc34-Δ12). It is known that Cdc34-Δ12 is a functional enzyme both *in vitro* and *in vivo* [19], [37], [38]. Moreover, the deletion of the acidic loop does not significantly change the overall structure of Cdc34 catalytic domain in a 100 ns MD simulations of Cdc34-Δ12^UBC^ (Figure 7 in Text S1). In a thiolester assay, Cdc34 migrates as a set of discrete bands, which are due to the linkage of multiple ubiquitin groups to lysine residues in Cdc34 protein. These autoubiquitination products depend upon the binding of the Ub molecule to the catalytic residue C95 [39], [40]. As previously reported [25], phosphorylation by CK2 strongly enhanced the ubiquitin-charging activity of His_6_-Cdc34 in a thiolester assay *in vitro*, mainly acting through S130 and S167 phosphorylations (Figure 9A). The Ub-charging activity of the mutant protein His_6_-Cdc34-Δ12 is unaffected by phosphorylation, since both the phosphorylated and unphosphorylated proteins are able to bind Ub to a comparable level (Figure 9A). To ascertain which of the many bands is the thiolester form, the samples of the thiolester assays were separated on both reducing and non reducing conditions (Figure 9B). The only species sensitive to DTT is the faster migrating band, corresponding to the Cdc34-Ub thiolester, as also reported by Banerjee et al. [39]. These results support the conclusions derived from MD investigation, indicating that the main role of CK2 phosphorylation on Cdc34^UBC^ is to promote the displacement to an open conformation of the acidic loop and to expose the catalytic cysteine to the solvent.

## Discussion

E2 enzymes define a complex superfamily that includes 17 families [14]. They play a major role in protein ubiquitination and Ub chain assembly [12], [13]. Recently, E2s have been shown to be involved in a variety of disorders, including cancers and neurodegenerative diseases [41], [42], [43]. Accordingly, there is increasing interest in understanding their regulation at the molecular level, a mandatory pre-requisite for rational design of effective pharmacologically active molecules which target E2 function.

An acidic insertion in the loop β2α4 has been identified in the proximity of the catalytic cysteine of several E2 enzymes, including Cdc34 that plays a major regulatory role in cell cycle progression and tumor development [41], [42]. The acidic loop has been thoroughly investigated by mutagenesis experiments. In fact, mutations of acid residues in the loop abolished the polyubiquitin chain assembly, the processivity and the synthesis of a polyubiquitin chain with a correct topology [22], [23], [24]. It has been proposed that the loop is crucial for E2 downstream signaling such as interactions with E3 or correct interactions with the target substrates. Nevertheless, the 12-residue acidic insertion is not essential for yeast Cdc34 function in over-expression conditions [37] and its role in Cdc34 function remains unexplained.

In this paper, we report that the loop acts as one element of a bipartite signature structure conserved among the Cdc34-like E2 enzymes belonging to family 3 and regulates enzyme activity through a phosphorylation mechanism. The second component of the signature structure is defined by serines within the catalytic domain, corresponding to S130 and S167 of yeast Cdc34, which are phosphorylated by CK2, a highly conserved protein kinase, essential in several different cellular processes. Together these two elements define a molecular switch that modulates opening and closing of the catalytic cleft, and whose dynamics can be finely regulated by CK2 phosphorylation (see below).

**Figure 10:**
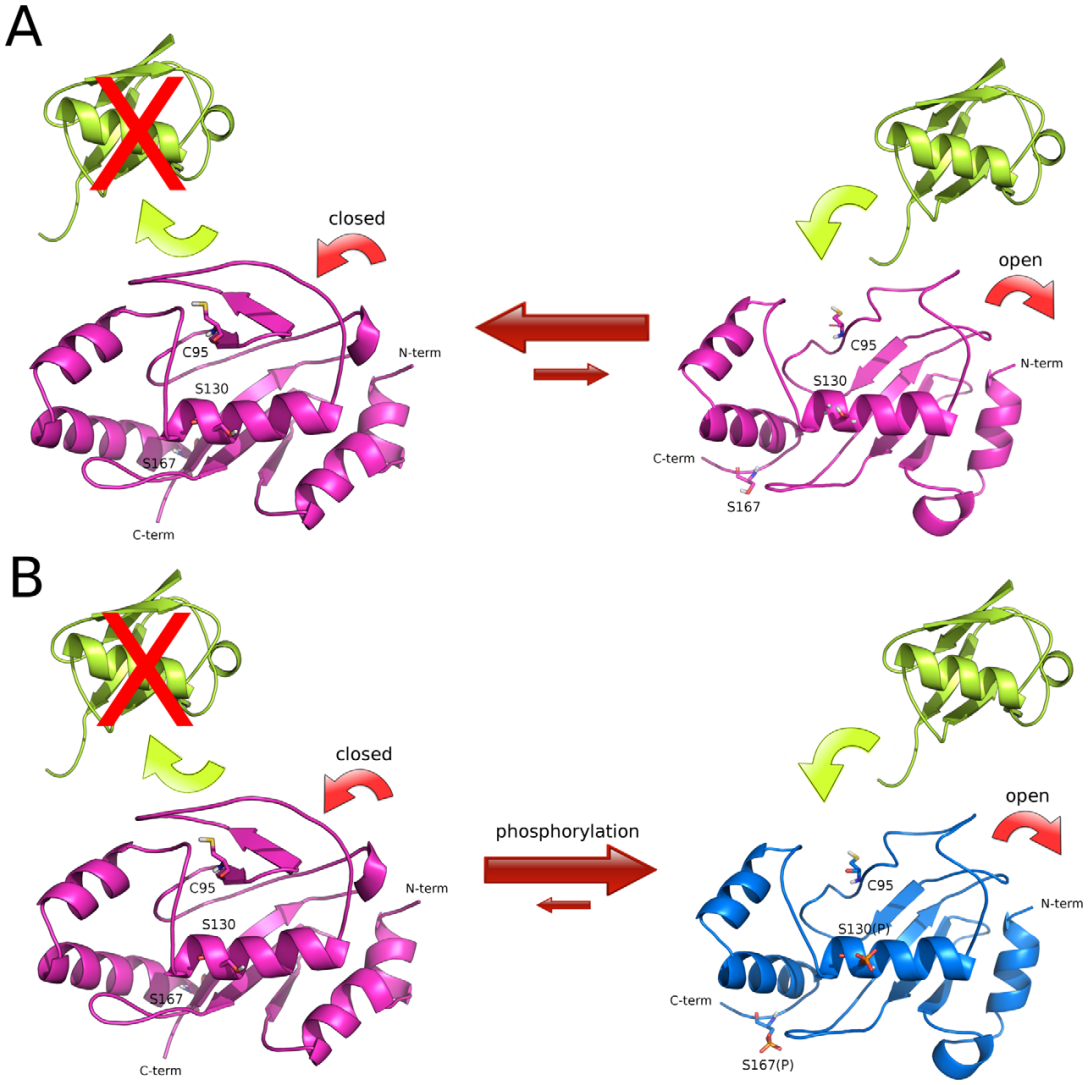
Model of the activation mechanism of ubiquitin charging activity of Cdc34-like E2 enzymes. Ubiquitin, unphosphorylated and phosphorylated Cdc34-like E2s are shown in yellow, magenta and blue, respectively. (A) In unphosphorylated E2 enzyme, the acidic loop can interconvert between open and closed conformations with respect to the catalytic site and ubiquitin charging is strongly disadvantaged. (B) Upon phosphorylation, the E2 enzyme is stabilized in an open conformation of the acidic loop, competent for ubiquitin charging, induced by electrostatic repulsion, which do not allow the closure of the loop on the catalytic cleft.

In the unphosphorylated protein, the β4α2 loop has high conformational freedom (Figure 4, 5A). MD trajectories show that acidic residues of the loop came close to S130 of Cdc34 located in an α-helix forming one side of the catalytic cleft (Figure 5-6). These movements make the loop act as a “lid”, switching from “open” to “closed” conformations with respect to the catalytic cleft (Figure 10). The mobility of the acidic loop in Cdc34^UBC^ and its modulation of solvent accessibility of the E2 catalytic cleft could explain the low basal Ub charging activity by unphosphorylated Cdc34 observed *in vitro* [25], also in agreement with the fact that the NMR structure of the human homolog Ube2g2, an E2 enzyme belonging to family 3, is characterized by conformations of the acidic insertion which in general does not provide accessibility of the catalytic cysteine [21].

Repulsive electrostatic effects between phospho-S130 and the acidic residues in the loop decrease its mobility, triggering an outward displacement of the loop and a competent conformation for Ub-charging (Figure 10). The proposed mechanism for Cdc34-like E2s activation is also in agreement with preliminary homology models of complexes between Cdc34-like enzymes and Ub, in which only open conformations of the loop are compatible with Ub interactions in the catalytic cleft (*data not shown*). It is in agreement as well with the previous observation that the acidic loop in a more closed conformation would result in steric clashes with the C-terminus of the ubiquitin molecule [36]. Our proposed model further predicts that if the electrostatic repulsive effects, which promote conformational changes of the loop, are abolished by mutations of the phospho-sites or of the acidic residues in the insertion, the loop cannot be stabilized in an open conformation upon phosphorylation, compromising downstream events in the Ub pathway. Consistently with this notion, while Cdc34 Ub-charging activity is modulated by CK2-dependent phosphorylation of S130 (and S167, see later) [25], ubiquitin charging activity of His_6_-Cdc34-Δ12 is unaffected by phosphorylation (Figure 9).

It has also to be considered, in our model, that ubiquitin-charging of E2 enzymes requires an interaction between E2 and E1 enzyme and a transfer of ubiquitin from the E1 catalytic cysteine to the E2 catalytic cysteine. In order, to strengthen our model, we derived by similarity with experimentally known E1-E2 complexes, a model of the putative complex between Uba1 E1 enzyme and Cdc34 with both open and closed conformations of the acidic loop (Figure 8 in Text S1). This qualitative model suggests that the acidic loop can be accommodated in the E1 binding cavity both in the open and closed conformation without causing steric effects, but if the loop is in a closed conformation it is likely to create a barrier between the E1 and E2 catalytic cysteines and probably prevents the transfer of Ub molecule. As it can be judged from the Cdc34-E1 model, the presence at position 130 of Cdc34 of serine or phospho-serine does not significantly affect the intermolecular interaction network at E1-E2 interface, even if further calculations will be necessary to clearly define the intermolecular interactions in details, whereas Uba1-Cdc34 model emphasizes the notion that the prominent role of S130 phosphorylation is to promote the acidic loop displacement (*data not shown*). Previous results showed that *in vivo* both S130 and S167 residues need to be mutated to alanine to make the yeast Cdc34 protein unable to complement a *cdc34-2^ts^* mutant, although evident morphological defects were observed in strains expressing Cdc34^S130A^ (but not Cdc34^S167A^) [25]. Phosphorylation of S130 residue is likely to account for the most relevant conformational changes induced by the post-translational modification, whereas S167 may have additive enhancing effects as suggested by double-phosphorylated Cdc34 MD simulations. In our model, S167 is located close to the C-terminal end. As a result, constraints on S167, as well as its interactions with other protein domains are likely to be lost. A detailed computational study of the role of S167 phosphorylation will require a suitable structure of the full-length Cdc34 protein.

In a broader context, it has been established that post-translational phosphorylation is a widespread mechanism for the regulation of protein biological activity [44], [45]. In several cases, enzyme activity or protein function has been reported to be regulated by inhibitory or activatory phosphorylation events at specific protein sites. At the molecular level, these events are mediated by electrostatic repulsion between the phospho-residues and the neighboring negatively charged aminoacids [46], [47], [48], [49], [50], [51], pointing out a general and important regulatory mechanism. These mechanisms can be successfully investigated in atomic details by MD simulations [47], [52].

In conclusion, our study sheds a new and unexpected light on the role of the acidic loop in Cdc34-like E2 enzymes and provides the first evidence that this loop is crucial not only for downstream events related to Ub chain assembly [20]–[22], but above all for modulation of an upstream crucial step of the Ub pathway: the covalent Ub-binding of Cdc34-like E2s. The loop activation by phosphorylation of serine residues in the UBC domain is a mandatory step for an efficient Ub-charging and could also account for the proper proceeding of downstream events in the ubiquitin pathway.

## Materials and Methods

### Homology modeling of yeast Cdc34 and alignments

Three different models have been generated with Modeller [53] for yeast Cdc34 UBC domain (7–170 residues, Cdc34^UBC^) using as templates the known X-ray structures of human Ube2g2 [PDB entry 2CYX [32]], yeast Ubc7 [PDB entry 2UCZ [33]] and *C. elegans* Ubc7 [PDB entry 1PZV (*to be published*)] enzymes, sharing with yeast Cdc34 more than 40% of sequence identity (Text S2).

Multiple sequence and structural alignments of 250 sequences of E2 UBC domains from each of the known 17 E2 families [13] have been performed by ClustalW [54] and DALI [55]. E2s from families 10, 16 and 17 have been discarded, as they are not catalytically active or characterized by a non canonical fold [13]. In particular, UBC domain sequences of each E2s have been determined by intra-family sequence alignments. For each family, a sequence alignment between Cdc34 and the members of the family has also been carried out (Figure 1 in 1).

### Molecular dynamics simulations

Molecular dynamics (MD) simulations were performed using GROMACS 3.3.3 (www.gromacs.org) with a modified version of GROMOS96 force field [56], [57]. The Cdc34 models (Cdc34^UBC^) and the 3D crystallographic structures of yeast Ubc7 and human Ube2g2, as well as of yeast Ubc1 [PDB entry 1FZY[15]] have been used as starting point for simulations. The structures were soaked in a dodechaedral box of SPC (Simple Point Charge) water molecules [58] in periodic boundary conditions (Text S2). In the simulations all the protein atoms were at a distance equal or greater than 0.6 nm from the box edges. Productive MD simulations were performed in the isothermal-isobaric (NPT) (300 K, 1 bar and 2 fs time-step). Electrostatic interactions were calculated using the Particle Mesh-Ewald (PME) summation scheme [59]. Van der Waals and Coulomb interactions were truncated at 1.0 nm and conformations stored every 4 ps. 4 independent simulations (replicas) were carried out for each non-phosphorylated Cdc34 model (Table 1), in order to improve the conformational sampling. MD multiple-replica approach has been shown to allow a wider conformational sampling than only few longer molecular dynamics simulations, if the stability of the trajectory and convergence of the analyzed properties have been carefully verified [60], [61], [62], [63]. The integration of structural cluster analysis and free energy landscape (Text S2, Figure 2 in Text S1) has been used in order to identify representative structures of Cdc34^UBC^ close to its native-state, according to a protocol previously published [64]. In particular, an iterative procedure was pursued, collecting independent replicas for the same protein system until parameters from principal component analysis reach convergence (see below) and results from cluster analysis and free energy landscape are congruent (Text S2, Figure 2 in Text S1).

The average structures from native Cdc34^UBC^ simulations have been used as starting structures for simulations of Cdc34^UBC^ mutant and phospho-variants. The replicas collected for each protein and mutant variant differ for duration and number (Table 1), allowing us to collect more than 2.5 µs of simulations.

MD simulations using the same approach have been also carried out on the two Cdc34 homologs yeast Ubc7 and human Ube2g2 for which known experimental structures are available. Moreover, since an NMR structure of Ube2g2 has been released during the manuscript preparation (PDB entry 2KLY [19]), control simulations have been also carried out starting from a model of Cdc34 using the Ube2g2 NMR structure as template, allowing to confirm the collected results (*data not shown*).

### Analysis of MD simulations

The rmsd (root mean square deviation) has been calculated with respect to the MD initial structures, both using all protein mainchain atoms (Figure 9 in Text S1) and mainchain atom of the common elements of the E2 fold (Figure 10 in Text S1). From 2 (Cdc34^UBC^ and Ubc7) to 10 ns (mutant and phospho-Cdc34^UBC^) of each replica are required for convergence. For each system, the equilibrated portions of each replica were joined in macro-trajectories, representative of different directions of sampling around the starting structure. In order to carefully checked rmsd profiles stability, sample simulations have been extended to 80/100 ns, indicating that the proteins are not still in a slow equilibration phase and that the resulting trajectories can be used to investigate dynamic and structural properties.

The secondary structure content was calculated by DSSP program (http://swift.cmbi.ru.nl/gv/dssp/). The root mean square fluctuation (rmsf) per residue from the average structure was calculated on α-carbons (Cα). In order to verify consistency of rmsf profiles, the Pearson correlation coefficient was calculated for rmsf data sets relative to replicas of the same system, collecting values higher than 0.6 (Text S2 for further details on MD analyses).

### Principal Component Analysis (PCA) and Free Energy Landscape (FEL)

PCA reveals high-amplitude concerted motion in MD trajectories, through eigenvectors of the Cα mass-weighted covariance matrix of atomic positional fluctuations [33] calculated for each replica and concatenated trajectories. In our concatenated trajectories the first 3 principal components (PCs) describe more than 60% of the total motion. Cosine content of the first 20 PCs has been calculated to evaluate the conformational sampling achieved by simulations (Text S2, Figure 11 in Text S1).

### Recombinant DNA techniques, genetic manipulations and purification of His_6_-Cdc34-Δ12

DNA manipulations and yeast transformations were carried out according to standard techniques. *E. coli DH5α* and *BL21(DE3)[pLysE]* were used in cloning experiments and for expression of recombinant proteins, respectively. *CDC34-Δ12* (containing CDC34 gene with the deletion of the sequence encoding aminoacids 103–114) was cloned in pIVEX2.4a plasmid using custom designed primers. His_6_-Cdc34-Δ12 recombinant protein was purified as previously reported [23].

### In vitro phosphorylation and thiolester assay

*In vitro* phosphorylation of recombinant proteins (7 µg of each purified protein) was performed as previously described [23], using 10 U of purified human CK2 (BIOMOL) in 10 mM Tris-HCl pH 7.5, 150 mM NaCl, 10 mM MgCl_2_, 100 µM ATP for 30 min at 30°C. CK2 phosphorylation was controlled using [γ-^32^P]-ATP. Both phosphorylated and not phosphorylated His_6_-Cdc34 and His_6_-Cdc34-Δ12 recombinant proteins were used in a thiolester assay. The reaction mixture contained the following in a volume of 40 µl: 50 mM Tris-HCl pH 7.5, 5 mM MgCl_2_, 2 mM DTT, 0.5 mM ATP, 20 ng/µl biotinylated-ubiquitin, 10 ng/µl E1 enzyme, 50 ng/µl His_6_-Cdc34 or His_6_-Cdc34-Δ12. After incubation at 37°C for the indicated time points, the reaction was stopped by the addition of sample buffer without β-mercaptoethanol (where indicated, 200 mM DTT was added). Samples were subjected to SDS-PAGE and analyzed by decoration with Horseradish-Peroxidase (HRP)-streptavidine.

## Supporting Information

Text S1 This file contains the following supporting figures for this article: Figure 1. Intra-family multiple sequence alignment. In each alignment the families are indicated by ‘_x’. Figure 2. Conformational landscape explored in Cdc34^UBC^ simulations. The free energy landscape is represented using projection of the Cdc34^UBC^ macro-trajectory along the principal components PC1 and PC2 of the essential subspace. The free energy is given in KJ/mol and indicated by the color bar. The label A–E indicates the region corresponding to the minimum free energy basins and the most populated structural clusters from cluster analysis. Figure 3 Flexibility profiles of non-phosphorylated Cdc34^UBC^. Cα rmsf of ensemble trajectories of Cdc34^UBC^ and Cdc34^UBC^–S130AS167A concatenated trajectories. The most persistent secondary structure during the simulations are represented schematically for each protein system. The rectangular box indicates the acidic loop. Figure 4. Flexibility profiles of phospho-Cdc34^UBC^–pS130pS167 simulations. Cα rmsf of ensemble trajectories of Cdc34^UBC^-pS130-pS167 and non-phosphorylated Cdc34^UBC^ are compared. The most persistent secondary structure during the simulations are represented schematically for each protein system. The rectangular box indicates the acidic loop. Figure 5. 3D structural superimposition of an average structure from phospho-Cdc34^UBC^simulations, free NMR and X-ray structure of Ube2g2 and Ube2g2 in complex with the gp78 region of its E3 partner. The average structure of phospho Cdc34^UBC^ simulations is shown in blue, the NMR (PDB entry 2KLY) and X-ray (PDB entry 2CYX) structure of Ube2g2 are shown in dark and light green, respectively, the structure of Ube2g2 in complex with gp78 region of E3 partner (PDB entry 3H8K) is shown in orange. The catalytic cysteine is shown as yellow stick. Figure 6. Projection of the simulations frames along the PC1 of Cdc34^UBC^-S130D concatenated trajectory, indicated with different shades of grey. The rectangular box indicates the acidic loop and its aminoacidic composition. Figure 7. Root mean square fluctuation (rmsf) profiles of Cdc34^UBC^ and Cdc34-Δ12^UBC^ domains. Figure 8. Model of Cdc34 both in closed (A) and open (B) conformations in complex with Uba1 E1 on the base of the known crystallographic structures of E2-E1 enzymes (PDB codes: 3CMM (Uba1) and 2PX9 (Ubc9 E2 in complex with SAE2 E1) and 2NVU (Ubc12 E2 in complex with Uba3 E1 and Nedd8). Figure 9. Mainchain root mean square deviation of single replicas of each simulated protein system. Figure 10. Mainchain root mean square deviation of the structural elements of the common E2-fold (the mainchain atoms of the acidic loop are not included in the analysis) of the Cdc34 simulations. Figure 11. Cosine content along the first 20 principal components of single replicas and concatenated trajectories of different length for each simulated protein system.
(PDF)

Text S2 Homology modeling of Cdc34^UBC^ to generate starting structures for molecular dynamics simulations and molecular dynamics simulations setup and analysis.
(PDF)
