# Supplementary material for: An Acidic Loop and Cognate Phosphorylation Sites Define a Molecular Switch That Modulates Ubiquitin Charging Activity in Cdc34-Like Enzymes
Source: PLoS Comput Biol. 2011 May 26;7(5):e1002056. doi: 10.1371/journal.pcbi.1002056 (PMC3102755; doi:10.1371/journal.pcbi.1002056)
Supplement: Text S1 — This file contains the following supporting figures for this article: Figure 1. Intra-family multiple sequence alignment. In each alignment the families are indicated by ‘_x’. Figure 2. Conformational landscape explored in Cdc34UBC simulations. The free energy landscape is represented using projection of the Cdc34UBC macro-trajectory along the principal components PC1 and PC2 of the essential subspace. The free energy is given in KJ/mol and indicated by the color bar. The label A–E indicates the region corresponding to the minimum free energy basins and the most populated structural clusters from cluster analysis. Figure 3 Flexibility profiles of non-phosphorylated Cdc34UBC. Cα rmsf of ensemble trajectories of Cdc34UBC and Cdc34UBC–S130AS167A concatenated trajectories. The most persistent secondary structure during the simulations are represented schematically for each protein system. The rectangular box indicates the acidic loop. Figure 4. Flexibility profiles of phospho-Cdc34UBC–pS130pS167 simulations. Cα rmsf of ensemble trajectories of Cdc34UBC-pS130-pS167 and non-phosphorylated Cdc34UBC are compared. The most persistent secondary structure during the simulations are represented schematically for each protein system. The rectangular box indicates the acidic loop. Figure 5. 3D structural superimposition of an average structure from phospho-Cdc34UBCsimulations, free NMR and X-ray structure of Ube2g2 and Ube2g2 in complex with the gp78 region of its E3 partner. The average structure of phospho Cdc34UBC simulations is shown in blue, the NMR (PDB entry 2KLY) and X-ray (PDB entry 2CYX) structure of Ube2g2 are shown in dark and light green, respectively, the structure of Ube2g2 in complex with gp78 region of E3 partner (PDB entry 3H8K) is shown in orange. The catalytic cysteine is shown as yellow stick. Figure 6. Projection of the simulations frames along the PC1 of Cdc34UBC-S130D concatenated trajectory, indicated with different shades of grey. The rectangular box ind [file pcbi.1002056.s001.pdf]

Figure 1. Intra-family multiple sequence alignment. In each alignment the families are indicated by '_x'.

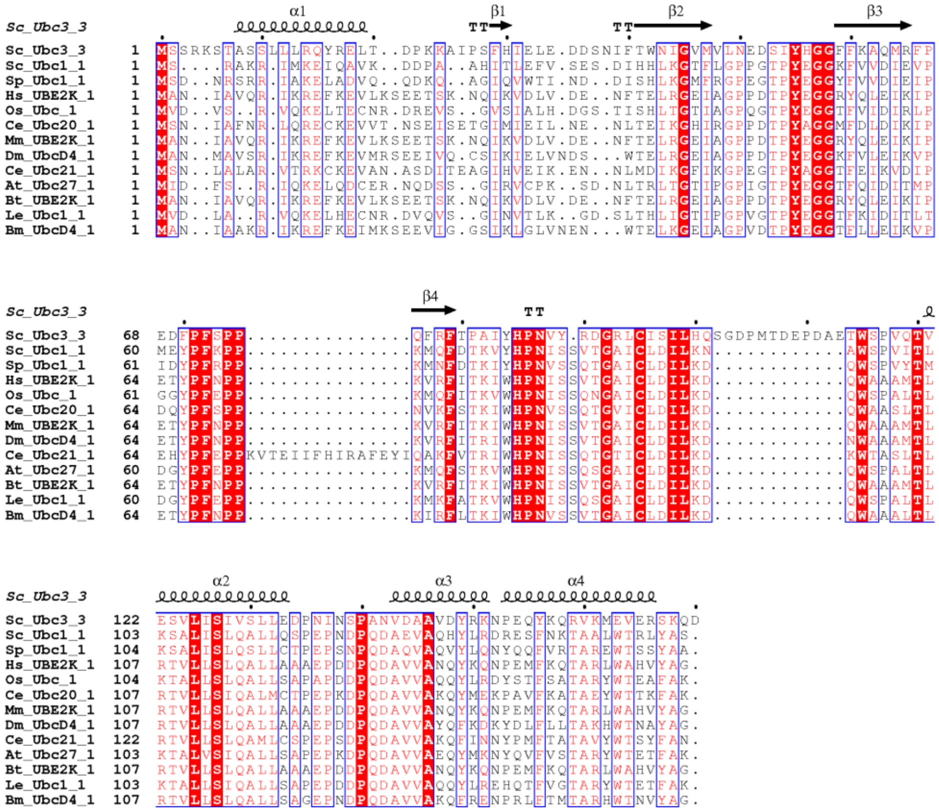

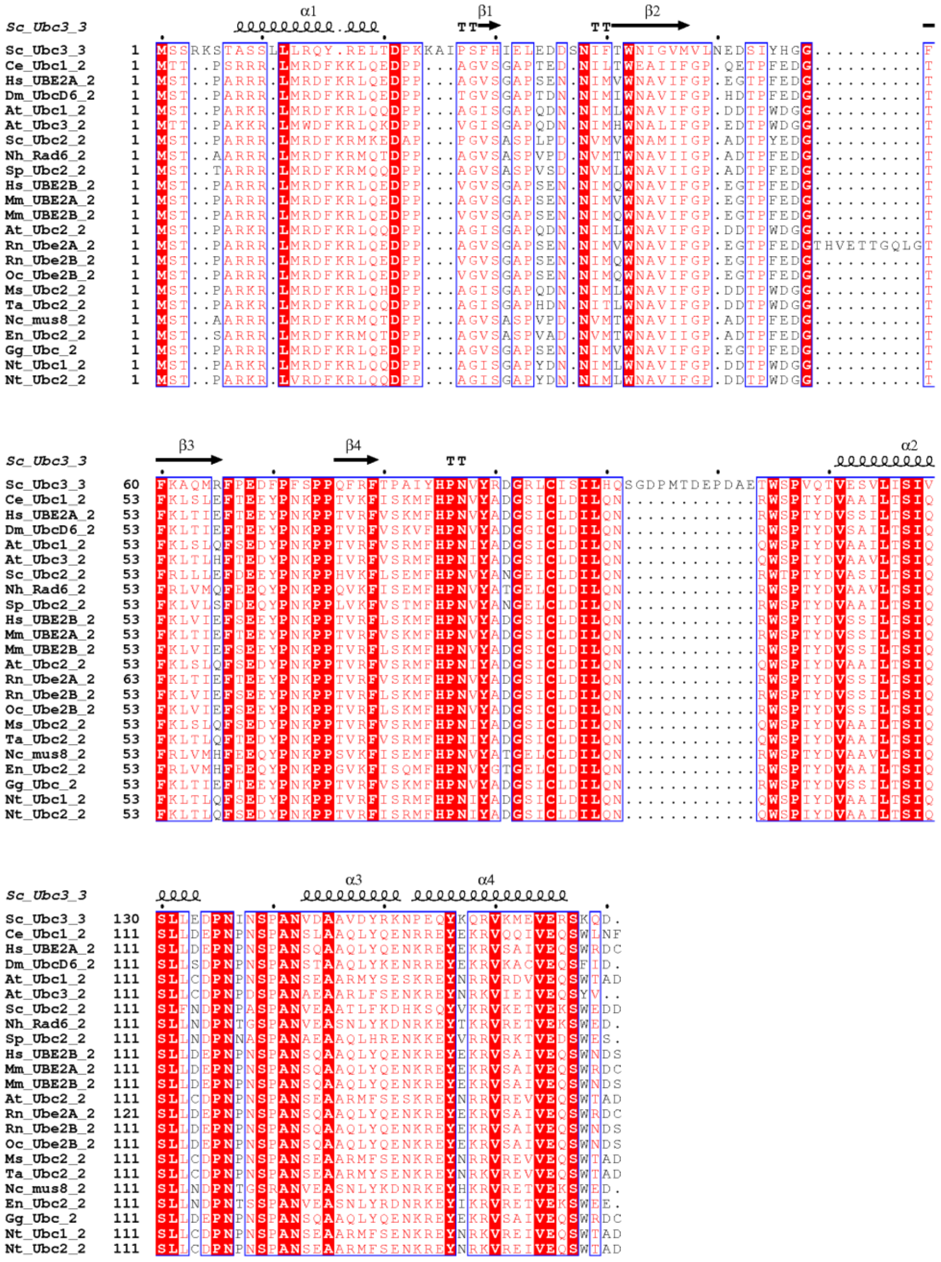
α1
β1
β2
β3
β4
α2
α3
α4
TT
Sc_Ubc3_3
Ce_Ubc1_2
Hs_UBE2A_2
Dm_UbcD6_2
At_Ubc1_2
At_Ubc3_2
Sc_Ubc2_2
Nh_Rad6_2
Sp_Ubc2_2
Hs_UBE2B_2
Mm_UBE2A_2
Mm_UBE2B_2
At_Ubc2_2
Rn_Ube2A_2
Rn_Ube2B_2
Oc_Ube2B_2
Ms_Ubc2_2
Ta_Ubc2_2
Nc_mus8_2
En_Ubc2_2
Gg_Ubc_2
Nt_Ubc1_2
Nt_Ubc2_2

```
                                      α1                 β1                  β2                  β3
Sc_Ubc3_3                  .    QQQQQQQQQQ.QQQ.     TT→   .         TT.→→→→→→→→→→→    .             →→→→→.→→→→→→
Sc_Ubc3_3        1   .MSSRKSTASSLLLRQY.RELTDPKKAIPSFHIELE..DDSNIFTWNIGVMVLNEDSIYHGGFFKAQMRF
Hs_Ubc2D1_4      1   ...MA...LKR.IQKELSDLQRDPPAH...CSAGPVG.DD..LFHWQATIMGP.PDSAYQGGVFFLTVHF
Hs_Ubc2D3_4      1   ...MA...LKR.INKELSDLARDPPAQ...CSAGPVG.DD..MFHWQATIMGP.NDSPYQGGVFFLTIHF
Hs_Ube2D4_4      1   ...MA...LKR.IQKELTDLQRDPPAQ...CSAGPVG.DD..LFHWQATIMGP.NDSPYQGGVFFLTIHF
Hs_UBE2E2_4      1   ..STSA...KR.IQKELAEITLDPPPN...CSAGPKG.DN..IYEWRSTILGP.PGSVYEGGVFFLDITF
Hs_UBE2E3_4      1   ..STSA...KR.IQKELAEITLDPPPN...CSAGPKG.DN..IYEWRSTILGP.PGSVYEGGVFFLDITF
Mm_Ube2D1_4      1   ...MA...LKR.IQKELSDLQRDPPAH...CSAGPVG.DD..LFHWQATIMGP.PDSAYQGGVFFLTVHF
Mm_Ube2D2_4      1   ...MA...LKR.IHKELNDLARDPPAQ...CSAGPVG.DD..MFHWQATIMGP.NDSPYQGGVFFLTIHF
Mm_Ube2D3_4      1   ...MA...LKR.INKELSDLARDPPAQ...CSAGPVG.DD..MFHWQATIMGP.NDSPYQGGVFFLTIHF
Mm_Ube2E1_4      1   ..STSA...KR.IQKELADITLDPPPN...CSAGPKG.DN..IYEWRSTILGP.PGSVYEGGVFFLDITF
Mm_Ube2E2_4      1   ..STSA...KR.IQKELAEITLDPPPN...CSAGPKG.DN..IYEWRSTILGP.PGSVYEGGVFFLDITF
Mm_Ube2E3_4      1   ..STSA...KR.IQKELAEITLDPPPN...CSAGPKG.DN..IYEWRSTILGP.PGSVYEGGVFFLDITF
Dm_UbcD1_4       1   ...MA...LKR.INKELQDLGRDPPAQ...CSAGPVG.DD..LFHWQATIMGP.PDSPYQGGVFFLTIHF
Dm_UbcD2_4       1   ..GTSA...KR.IQKELAEITLDPPPN...CSAGPKG.DN..LYEWVSTILGP.PGSVYEGGVFFLDIHF
Sc_Ubc5_4        1   ...MSS..SKR.IAKELSDLGRDPPAS...CSAGPVG.DD..LYHWQASIMGP.SDSPYAGGVFFLSIHF
Sp_Ubc4_4        1   ...MA...LKR.INRELADLGKDPPSS...CSAGPVG.DD..LFHWQATIMGP.ADSPYAGGVFFLSIHF
Sp_Ubc14_4       1   MASASPSSSRR.LTKEYSDLREHPIPD...IRVNLVD.DN..LFHWACTALGP.SDSVYAGGKFHFSLKF
At_Ubc30_4       1   ...MA...SKR.INKELRDLQRDPPVS...CSAGPTG.DD..MFQWQATIMGP.ADSPFAGGVFLVTIHF
At_Ubc8_4        1   ...MA...SKR.ILKELKDLQKDPPTS...CSAGPVA.ED..MFHWQATIMGP.AESPYSGGVFLVTIHF
At_Ubc9_4        1   ...MA...SKR.ILKELKDLQKDPPTS...CSAGPVA.ED..MFHWQATIMGP.SDSPYSGGVFLVTIHF
At_Ubc10_4       1   ...MA...SKR.ILKELKDLQKDPPTS...CSAGPVA.ED..MFHWQATIMGP.SESPYAGGVFLVTIHF
At_Ubc11_4       1   ...MA...SKR.ILKELKDLQKDPPSN...CSAGPVA.ED..MFHWQATIMGP.PESPYAGGVFLVSIHF
At_Ubc12_4       1   ...MA...SKR.ISRELRDMQRHPPAN...CSAGPVAEED..IFHWQATIMGP.HDSPYSGGVFTVSIDF
At_Ubc28_4       1   ...MA...SKR.ILKELKDLQKDPPTS...CSAGPVA.ED..MFHWQATIMGP.SDSPYSGGVFLVTIHF
At_Ubc29_4       1   ...MA...TRR.ILKELKELQRDPPVS...CSAGPTG.ED..MFHWQATIMGP.NESPYSGGVFLVNIHF
Le_Ubc4_4        1   ...MA...SKR.ILKELKDLQKDPPTS...CSAGPVA.ED..MFHWQATIMGP.TDSPYAGGVFLVSIHF
Os_Ubc_4         1   ...MA...SKR.ILFCLKCTPKDPPTS...CSAGPVA.ED..MFHWQATIMGP.PDSPYAGGVFLVSIHF
Dd_UbcB_4        1   ...MAA..HKR.LQKEITDMLKTPPSW...CSAHLVD.DN..LQKWKATVQGP.EGSPFEKGVFSMDIDI
Cg_Ubc1_4        1   ...MA...FKR.INKELTDLGRDPPSS...CSAGPVG.ED..LFHWQATIMGP.GDSPYSGGVFFLAIHF
Mg_Ubc1_4        1   ...MA...LKR.INKELTDLGRDPPSS...CSAGPVG.ED..LFHWQATIMGP.SDSPYAGGVFFLAIHF
Rn_Ube2d2_4      1   ...MA...LKR.IHKELNDLARDPPAQ...CSAGPVG.DD..MFHWQATIMGP.NDSPYQGGVFFLTIHF
Rn_Ube2d2b_4     1   ...MA...LKR.IHKELNDLAQDPPAQ...CSAGPVG.ED..MFHWQATIMGP.NDSPYQGGAFFLTIDF
Rn_Ube2d3_4      1   ...MA...LKR.INKELSDLARDPPAQ...CSAGPVG.DD..MFHWQATIMGP.NDSPYQGGVFFLTIHF
Bt_Ube2d3_4      1   ...MA...LKR.INKELSDLARDPPAQ...CSAGPVG.DD..MFHWQATIMGP.NDSPYQGGVFFLTIHF
Pr_Ubc_4         1   ...MA...AKR.ITKELQDLQRDPPIS...CSTGPVG.DD..LFHWQATIMGP.VDSPYAGGVFFIVIHF
Zm_Ubc_4         1   ...MA...SKR.ILKELKDLQKDPPTS...CSAGPAG.ED..MFHWQATIMGP.PDSPYAGGVFLVNIHF
Le_Ubc_4         1   ...MA...SKR.ILKELKDLQKDPPTS...CSAGPVA.ED..MFHWQATIMGP.ADSPYSGGVFLVTIHF
Os_Ubc5a_4       1   ...MA...SKR.IQKELKDLQKDPPTS...CSAGPVG.ED..MFHWQATIMGP.SDSPYAGGVFLVTIHF
Ca_Ubc4_4        1   ...MS...LKR.INKELSDLGRDPPSS...CSAGPVG.DD..LYHWQASIMGP.PDSPYAGGVFFLSIHF
Ps_Ubc4_4        1   ...MA...SKR.ILKELKDLQKDPPTS...CSAGPVA.ED..MFHWQATIVGP.ADSPYAGGVFLVTIHF
Hs_UBE2U_4       1   ...MHGRAYLL.LHRDFCDLKENNY...KGITAKPVS.ED..MMEWEVEIEGL.QNSVWQGLVFQLTIHF
Hs_UBE2E1_4      1   ..STSA...KR.IQKELADITLDPPPN...CSAGPKG.DN..IYEWRSTILGP.PGSVYEGGVFFLDITF
Sc_Ubc4_4        1   ...MSS..SKR.IAKELSDLERDPPTS...CSAGPVG.DD..LYHWQASIMGP.ADSPYAGGVFFLSIHF
Ce_Ubc2_4        1   ...MA...LKR.IQKELQDLGRDPPAQ...CSAGPVG.DD..LFHWQATIMGP.PESPYQGGVFFLTIHF
Xl_Ubc4_4        1   ...MA...LKR.IHKELNDLARDPPAQ...CSAGPVG.DD..MFHWQATIMGP.NDSPYQGGVFFLTIHF
Hs_Ubc2D2_4      1   ...MA...LKR.IHKELNDLARDPPAQ...CSAGPVG.DD..MFHWQATIMGP.NDSPYQGGVFFLTIHF
Mm_MGC58426_4    1   ...MA...LKR.IQKELVAISQDPPAH...CSAGPVA.EN..MFHWQATIMGP.EDSPYQGGVFFLSVHF
Dm_CG5440_4      1   TCSNSA..VKR.IQKELDEITRDPPQY...CSAGPKE.DN..LYEWTSTIIGP.ADSVYENGIFKLDIFF
Dm_CG10862_4     1   ..PLT...ITR.LRREISEFSTDQTEG...CKAEMVG.DN..LFHWVATIPGP.SETVYEGGRFRVEIVF
Dm_CG2574_4      1   ...TGC..VVR.IKSELQDIRKNPPPN...CTADLHH.GD..LLHWTAGVNGP.VGSVYEGGHFRLDIRF
```

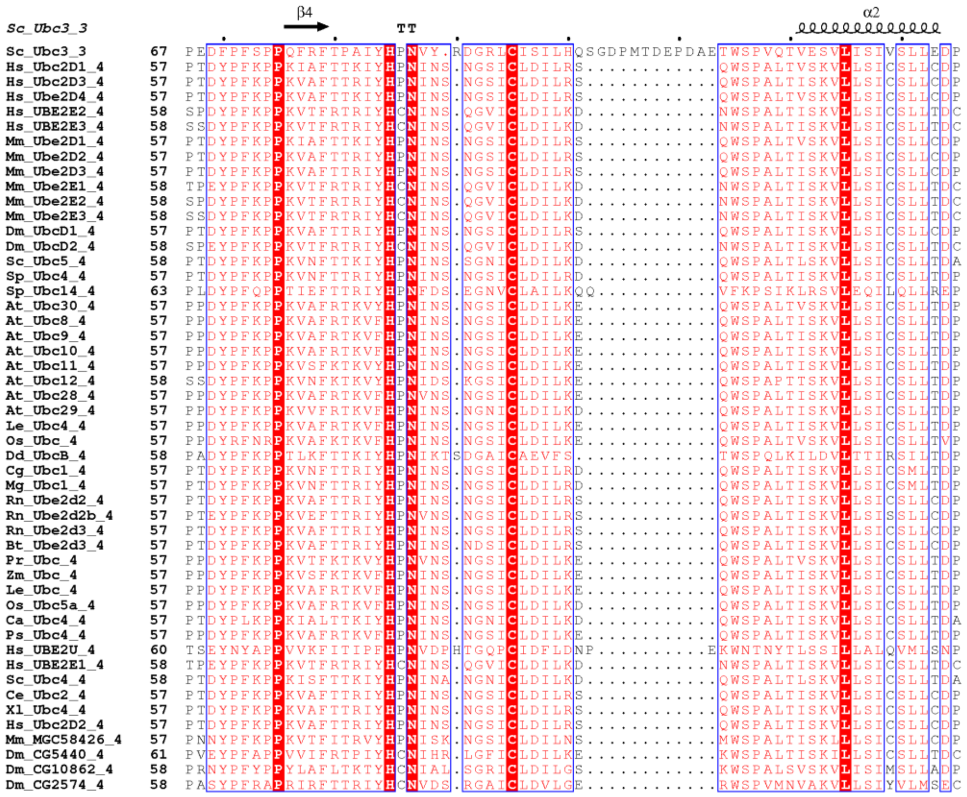
β4
TT
α2
Sc_Ubc3_3
Sc_Ubc3_3 67 PEDFPFSPPQFRFTPAIYHPNVY.RDGRLCISILHQSGDPMTDEPDAETWSPVQTVESVLISIVSLLEDP
Hs_Ubc2D1_4 57 PTDYPFKPPKIAFTTKIYHPNINS.NGSICLDILRS............QWSPALTVSKVLLSICSLLCDP
Hs_Ubc2D3_4 57 PTDYPFKPPKVAFTTRIYHPNINS.NGSICLDILRS............QWSPALTISKVLLSICSLLCDP
Hs_Ube2D4_4 57 PTDYPFKPPKVAFTTKIYHPNINS.NGSICLDILRS............QWSPALTVSKVLLSICSLLCDP
Hs_UBE2E2_4 58 SPDYPFKPPKVTFRTRIYHCNINS.QGVICLDILKD............NWSPALTISKVLLSICSLLTDC
Hs_UBE2E3_4 58 SSDYPFKPPKVTFRTRIYHCNINS.QGVICLDILKD............NWSPALTISKVLLSICSLLTDC
Mm_Ube2D1_4 57 PTDYPFKPPKIAFTTKIYHPNINS.NGSICLDILRS............QWSPALTVSKVLLSICSLLCDP
Mm_Ube2D2_4 57 PTDYPFKPPKVAFTTRIYHPNINS.NGSICLDILRS............QWSPALTISKVLLSICSLLCDP
Mm_Ube2D3_4 57 PTDYPFKPPKVAFTTRIYHPNINS.NGSICLDILRS............QWSPALTISKVLLSICSLLCDP
Mm_Ube2E1_4 58 TPEYPFKPPKVTFRTRIYHCNINS.QGVICLDILKD............NWSPALTISKVLLSICSLLTDC
Mm_Ube2E2_4 58 SPDYPFKPPKVTFRTRIYHCNINS.QGVICLDILKD............NWSPALTISKVLLSICSLLTDC
Mm_Ube2E3_4 58 SSDYPFKPPKVTFRTRIYHCNINS.QGVICLDILKD............NWSPALTISKVLLSICSLLTDC
Dm_UbcD1_4 57 PTDYPFKPPKVAFTTRIYHPNINS.NGSICLDILRS............QWSPALTISKVLLSICSLLCDP
Dm_UbcD2_4 58 SPEYPFKPPKVTFRTRIYHCNINS.QGVICLDILKD............NWSPALTISKVLLSICSLLTDC
Sc_Ubc5_4 58 PTDYPFKPPKVNFTTKIYHPNINS.SGNICLDILKD............QWSPALTLSKVLLSICSLLTDA
Sp_Ubc4_4 57 PTDYPFKPPKVNFTTRIYHPNINS.NGSICLDILRD............QWSPALTISKVLLSICSLLTDP
Sp_Ubc14_4 63 PLDYPFQPPTIEFTTRIYHPNFDS.EGNVCLAILKQQ...........VFKPSIKLRSVLEQILQLLREP
At_Ubc30_4 57 PPDYPFKPPKVAFRTKVYHPNINS.NGSICLDILKE............QWSPALTVSKVLLSICSLLTDP
At_Ubc8_4 57 PPDYPFKPPKVAFRTKVFHPNINS.NGSICLDILKE............QWSPALTISKVLLSICSLLTDP
At_Ubc9_4 57 PPDYPFKPPKVAFRTKVFHPNINS.NGSICLDILKE............QWSPALTISKVLLSICSLLTDP
At_Ubc10_4 57 PPDYPFKPPKVAFRTKVFHPNINS.NGSICLDILKE............QWSPALTISKVLLSICSLLTDP
At_Ubc11_4 57 PPDYPFKPPKVSFKTKVYHPNINS.NGSICLDILKE............QWSPALTISKVLLSICSLLTDP
At_Ubc12_4 58 SSDYPFKPPKVNFKTKVYHPNIDS.KGSICLDILKE............QWSPAPTTSKVLLSICSLLTDP
At_Ubc28_4 57 PPDYPFKPPKVAFRTKVFHPNVNS.NGSICLDILKE............QWSPALTISKVLLSICSLLTDP
At_Ubc29_4 57 PPDYPFKPPKVVFRTKVFHPNINS.NGNICLDILKD............QWSPALTISKVLLSICSLLTDP
Le_Ubc4_4 57 PPDYPFKPPKVAFRTKVFHPNINS.NGSICLDILKE............QWSPALTISKVLLSICSLLTDP
Os_Ubc_4 57 PPDYRFNRPKVAFKTKVFHPNINS.NGSICLDILKE............QWSPALTVSKVLLSICSLLTVP
Dd_UbcB_4 58 PADYPFKPPTLKFTTKIYHPNIKTSDGAICAEVFS.............TWSPQLKILDVLTTIRSILTDP
Cg_Ubc1_4 57 PTDYPFKPPKVNFTTRIYHPNINS.NGSICLDILRD............QWSPALTISKVLLSICSMLTDP
Mg_Ubc1_4 57 PTDYPFKPPKVNFTTRIYHPNINS.NGSICLDILRD............QWSPALTISKVLLSICSMLTDP
Rn_Ube2d2_4 57 PTDYPFKPPKVAFTTRIYHPNINS.NGSICLDILRS............QWSPALTISKVLLSICSLLCDP
Rn_Ube2d2b_4 57 PTEYPFKPPKVEFTTRIYHPNVNS.NGSICLDILRS............QWSPALTISKVLLSISSLLCDP
Rn_Ube2d3_4 57 PTDYPFKPPKVAFTTRIYHPNINS.NGSICLDILRS............QWSPALTISKVLLSICSLLCDP
Bt_Ube2d3_4 57 PTDYPFKPPKVAFTTRIYHPNINS.NDSICLDILRS............QWSPALTISKVLLSICSLLCDP
Pr_Ubc_4 57 PPDYPFKPPKVTFKTKVYHPNVNS.NGSICLDILKE............QWSPALTVSKVLLSICSLLTDP
Zm_Ubc_4 57 PPDYPFKPPKVSFKTKVFHPNINS.NGSICLDILKE............QWSPALTISKVLLSICSLLTDP
Le_Ubc_4 57 PPDYPFKPPKVAFRTKVFHPNINS.NGSICLDILKE............QWSPALTISKVLLSICSLLTDP
Os_Ubc5a_4 57 PPDYPFKPPKVAFRTKVFHPNINS.NGSICLDILKD............QWSPALTISKVLLSICSLLTDP
Ca_Ubc4_4 57 PTDYPLKPPKIALTTKIYHPNINS.NGNICLDILKD............QWSPALTISKVLLSICSLLTDA
Ps_Ubc4_4 57 PPDYPFKPPKVAFRTKVFHPNINS.NGSICLDILKE............QWSPALTISKVLLSICSLLTDP
Hs_UBE2U_4 60 TSEYNYAPPVVKFITIPFHPNVDPHTGQPCIDFLDNP..........EKWNTNYTLSSILLALQVMLSNP
Hs_UBE2E1_4 58 TPEYPFKPPKVTFRTRIYHCNINS.QGVICLDILKD............NWSPALTISKVLLSICSLLTDC
Sc_Ubc4_4 58 PTDYPFKPPKISFTTKIYHPNINA.NGNICLDILKD............QWSPALTLSKVLLSICSLLTDA
Ce_Ubc2_4 57 PTDYPFKPPKVAFTTRIYHPNINS.NGSICLDILRS............QWSPALTISKVLLSICSLLCDP
Xl_Ubc4_4 57 PTDYPFKPPKVAFTTRIYHPNINS.NGSICLDILRS............QWSPALTISKVLLSICSLLCDP
Hs_Ubc2D2_4 57 PTDYPFKPPKVAFTTRIYHPNINS.NGSICLDILRS............QWSPALTISKVLLSICSLLCDP
Mm_MGC58426_4 57 PNNYPFKPPKVTFITRVYHPNISK.NGSICLDILNS............MWSPALTISKLLLSICSLLCDP
Dm_CG5440_4 61 PVEYPFAPPVVIFRTPIYHCNIHR.LGFICLDILKE............KWSPALTISKILLSICSLLTDC
Dm_CG10862_4 58 PRNYPFYPPYLAFLTKTYHCNIAL.SGRICLDILGS............KWSPALSVSKVLISIMSLLADP
Dm_CG2574_4 58 PASYPFRAPRIRFTTRIYHCNVDS.RGAICLDVLGE............RWSPVMNVAKVLLSIYVLMSEC

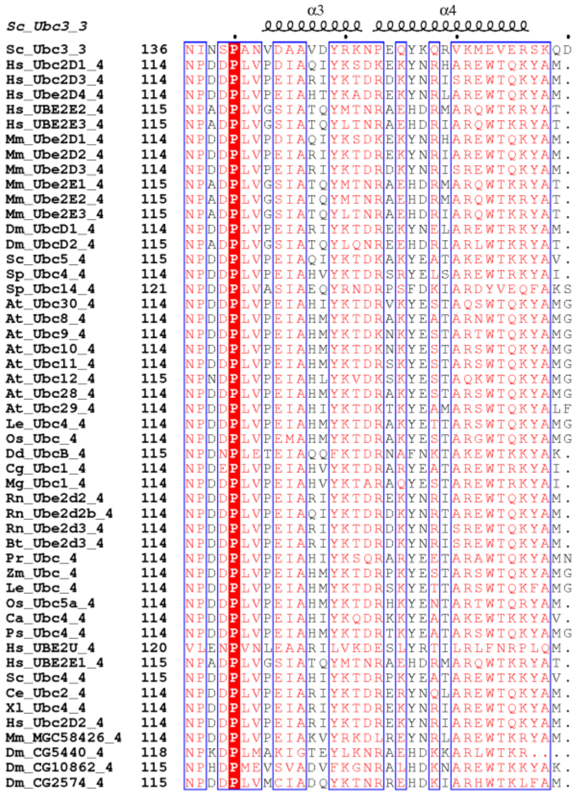
α3
α4
Sc_Ubc3_3
Sc_Ubc3_3 136 NINSPANVDAAVDYRKNPEQYKQRVKMEVERSKQD
Hs_Ubc2D1_4 114 NPDDPLVPDIAQIYKSDKEKYNRHAREWTQKYAM.
Hs_Ubc2D3_4 114 NPDDPLVPEIARIYKTDRDKYNRISREWTQKYAM.
Hs_Ube2D4_4 114 NPDDPLVPEIAHTYKADREKYNRLAREWTQKYAM.
Hs_UBE2E2_4 115 NPADPLVGSIATQYMTNRAEHDRMARQWTKRYAT.
Hs_UBE2E3_4 115 NPADPLVGSIATQYLTNRAEHDRIARQWTKRYAT.
Mm_Ube2D1_4 114 NPDDPLVPDIAQIYKSDKEKYNRHAREWTQKYAM.
Mm_Ube2D2_4 114 NPDDPLVPEIARIYKTDREKYNRIAREWTQKYAM.
Mm_Ube2D3_4 114 NPDDPLVPEIARIYKTDRDKYNRISREWTQKYAM.
Mm_Ube2E1_4 115 NPADPLVGSIATQYMTNRAEHDRMARQWTKRYAT.
Mm_Ube2E2_4 115 NPADPLVGSIATQYMTNRAEHDRMARQWTKRYAT.
Mm_Ube2E3_4 115 NPADPLVGSIATQYLTNRAEHDRIARQWTKRYAT.
Dm_UbcD1_4 114 NPDDPLVPEIARIYKTDREKYNELAREWTRKYAM.
Dm_UbcD2_4 115 NPADPLVGSIATQYLQNREEHDRIARLWTKRYAT.
Sc_Ubc5_4 115 NPDDPLVPEIAQIYKTDKAKYEATAKEWTKKYAV.
Sp_Ubc4_4 114 NPDDPLVPEIAHVYKTDRSRYELSAREWTRKYAI.
Sp_Ubc14_4 121 NPDDPLVASIAEQYRNDRPSFDKIARDYVEQFAKS
At_Ubc30_4 114 NPDDPLVPEIAHIYKTDRVKYESTAQSWTQKYAMG
At_Ubc8_4 114 NPDDPLVPEIAHMYKTDRAKYEATARNWTQKYAMG
At_Ubc9_4 114 NPDDPLVPEIAHMYKTDKNKYESTARTWTQKYAMG
At_Ubc10_4 114 NPDDPLVPEIAHMYKTDKNKYESTARSWTQKYAMG
At_Ubc11_4 114 NPDDPLVPEIAHMYKTDRSKYESTARSWTQKYAMG
At_Ubc12_4 115 NPNDPLVPEIAHLYKVDKSKYESTAQKWTQKYAMG
At_Ubc28_4 114 NPDDPLVPEIAHMYKTDRAKYESTARSWTQKYAMG
At_Ubc29_4 114 NPDDPLVPEIAHIYKTDKTKYEAMARSWTQKYALF
Le_Ubc4_4 114 NPDDPLVPEIAHMYKTDRAKYETTARSWTQKYAMG
Os_Ubc_4 114 NPDDPLVPEMAHMYKTDRAKYESTARGWTQKYAMG
Dd_UbcB_4 115 NPDNPLETEIAQQFKTDRNAFNKTAKEWTKKYAK.
Cg_Ubc1_4 114 NPDEPLVPEIAHVYKTDRARYEATAREWTRKYAI.
Mg_Ubc1_4 114 NPDDPLVPEIAHVYKTARAQYESTAREWTRKYAI.
Rn_Ube2d2_4 114 NPDDPLVPEIARIYKTDREKYNRIAREWTQKYAM.
Rn_Ube2d2b_4 114 NPDDPLVPEIAQIYKTDRDKYNRTAREWTQKYAM.
Rn_Ube2d3_4 114 NPDDPLVPEIARIYKTDRDKYNRISREWTQKYAM.
Bt_Ube2d3_4 114 NPDDPLVPEIARIYKTDRDKYNRISREWTQKYAM.
Pr_Ubc_4 114 NPDDPLVPEIAHIYKSQRARYEETARAWTQKYAMN
Zm_Ubc_4 114 NPDDPLVPEIAHMYKTDRPKYESTARSWTQKYAMG
Le_Ubc_4 114 NPDDPLVPEIAHMYKTDRSKYETTARSWTQKFAMG
Os_Ubc5a_4 114 NPDDPLVPEIAHMYKTDRHKYENTARTWTQRYAM.
Ca_Ubc4_4 114 NPDDPLVPEIAHIYKQDRKKYEATAKEWTKKYAV.
Ps_Ubc4_4 114 NPDDPLVPEIAHMYKTDRTKYEATARSWTQKYAMG
Hs_UBE2U_4 120 VLENPVNLEAARILVKDESLYRTILRLFNRPLQM.
Hs_UBE2E1_4 115 NPADPLVGSIATQYMTNRAEHDRMARQWTKRYAT.
Sc_Ubc4_4 115 NPDDPLVPEIAHIYKTDRPKYEATAREWTKKYAV.
Ce_Ubc2_4 114 NPDDPLVPEIARIYKTDRERYNQLAREWTQKYAM.
Xl_Ubc4_4 114 NPDDPLVPEIARIYKTDREKYNRIAREWTQKYAM.
Hs_Ubc2D2_4 114 NPDDPLVPEIARIYKTDREKYNRIAREWTQKYAM.
Mm_MGC58426_4 114 NPDDPLVPEIAKVYRKDLREYNRLAREWTKRYAM.
Dm_CG5440_4 118 NPKDPLMAKIGTEYLKNRAEHDKKARLWTKR....
Dm_CG10862_4 115 NPHDPMEVSVADVFKGNRALHDKNAREWTKKYAK.
Dm_CG2574_4 115 NPDDPLVMCIADQYKTNRREHDKIARHWTKLFAM.

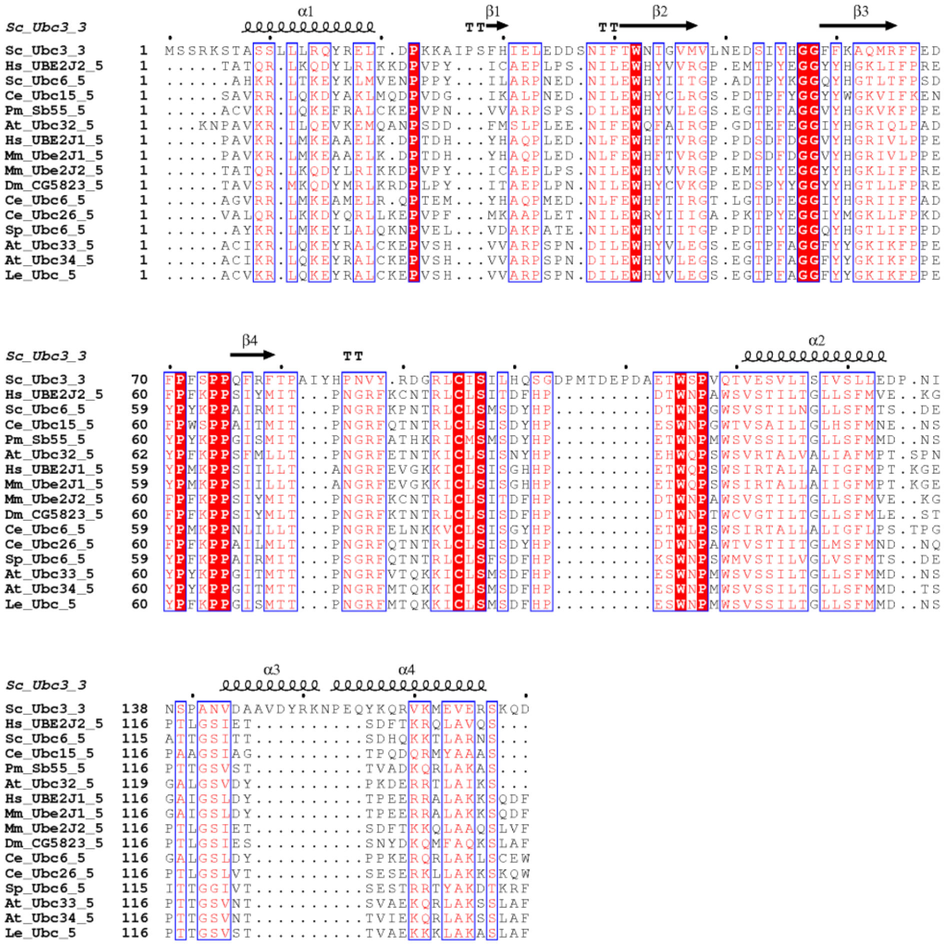
α1 β1 β2 β3
TT TT
Sc_Ubc3_3
Sc_Ubc3_3 1 MSSRKSTASSLLLRQYRELT.DPKKAIPSFHIELEDDSNIFTWNIGVMVLNEDSIYHGGFFKAQMRFPED
Hs_UBE2J2_5 1 .....TATQR.LKQDYLRIKKDPVPY...ICAEPLPS.NILEWHYVVRGP.EMTPYEGGYYHGKLIFPRE
Sc_Ubc6_5 1 ......AHKR.LTKEYKLMVENPPPY...ILARPNED.NILEWHYIITGP.ADTPYKGGQYHGTLTFPSD
Ce_Ubc15_5 1 .....SAVRR.LQKDYAKLMQDPVDG...IKALPNED.NILEWHYCLRGS.PDTPFYGGYYWGKVIFKEN
Pm_Sb55_5 1 .....ACVKR.LQKEFRALCKEPVPN...VVARPSPS.DILEWHYVLEGS.EGTPFAGGVYHGKVKFPPE
At_Ubc32_5 1 ...KNPAVKR.ILQEVKEMQANPSDD...FMSLPLEE.NIFEWQFAIRGP.GDTEFEGGIYHGRIQLPAD
Hs_UBE2J1_5 1 .....PAVKR.LMKEAAELK.DPTDH...YHAQPLED.NLFEWHFTVRGP.PDSDFDGGVYHGRIVLPPE
Mm_Ube2J1_5 1 .....PAVKR.LMKEAAELK.DPTDH...YHAQPLED.NLFEWHFTVRGP.PDSDFDGGVYHGRIVLPPE
Mm_Ube2J2_5 1 .....TATQR.LKQDYLRIKKDPVPY...ICAEPLPS.NILEWHYVVRGP.EMTPYEGGYYHGKLIFPRE
Dm_CG5823_5 1 .....TAVSR.MKQDYMRLKRDPLPY...ITAEPLPN.NILEWHYCVKGP.EDSPYYGGYYHGTLLFPRE
Ce_Ubc6_5 1 .....AGVRR.LMKEAMELR.QPTEM...YHAQPMED.NLFEWHFTIRGT.LGTDFEGGIYHGRIIFPAD
Ce_Ubc26_5 1 .....VALQR.LKKDYQRLLKEPVPF...MKAAPLET.NILEWRYIIIGA.PKTPYEGGIYMGKLLFPKD
Sp_Ubc6_5 1 ......AYKR.LMKEYLALQKNPVEL...VDAKPATE.NILEWHYIITGP.PDTPYEGGQYHGTLIFPPD
At_Ubc33_5 1 .....ACIKR.LQKEYRALCKEPVSH...VVARPSPN.DILEWHYVLEGS.EGTPFAGGFYYGKIKFPPE
At_Ubc34_5 1 .....ACIKR.LQKEYRALCKEPVSH...VVARPSPN.DILEWHYVLEGS.EGTPFAGGFYYGKIKFPPE
Le_Ubc_5 1 .....ACVKR.LQKEYRALCKEPVSH...VVARPSPN.DILEWHYVLEGS.EGTPFAGGFYYGKIKFPPE
β4 α2
TT
Sc_Ubc3_3
Sc_Ubc3_3 70 FPFSPPQFRFTPAIYHPNVY.RDGRLCISILHQSGDPMTDEPDAETWSPVQTVESVLISIVSLLEDP.NI
Hs_UBE2J2_5 60 FPFKPPSIYMIT...PNGRFKCNTRLCLSITDFHP.........DTWNPAWSVSTILTGLLSFMVE..KG
Sc_Ubc6_5 59 YPYKPPAIRMIT...PNGRFKPNTRLCLSMSDYHP.........DTWNPGWSVSTILNGLLSFMTS..DE
Ce_Ubc15_5 60 FPWSPPAITMIT...PNGRFQTNTRLCLSISDYHP.........ESWNPGWTVSAILIGLHSFMNE..NS
Pm_Sb55_5 60 YPYKPPGISMIT...PNGRFATHKRICMSMSDYHP.........ETWNPMWSVSSILTGLLSFMMD..NS
At_Ubc32_5 62 YPFKPPSFMLLT...PNGRFETNTKICLSISNYHP.........EHWQPSWSVRTALVALIAFMPT.SPN
Hs_UBE2J1_5 59 YPMKPPSIILLT...ANGRFEVGKKICLSISGHHP.........ETWQPSWSIRTALLAIIGFMPT.KGE
Mm_Ube2J1_5 59 YPMKPPSIILLT...ANGRFEVGKKICLSISGHHP.........ETWQPSWSIRTALLAIIGFMPT.KGE
Mm_Ube2J2_5 60 FPFKPPSIYMIT...PNGRFKCNTRLCLSITDFHP.........DTWNPAWSVSTILTGLLSFMVE..KG
Dm_CG5823_5 60 FPFKPPSIYMLT...PNGRFKTNTRLCLSISDFHP.........DTWNPTWCVGTILTGLLSFMLE..ST
Ce_Ubc6_5 59 YPMKPPNLILLT...PNGRFELNKKVCLSISGYHP.........ETWLPSWSIRTALLALIGFLPS.TPG
Ce_Ubc26_5 60 FPFKPPAILMLT...PNGRFQTNTRLCLSISDYHP.........DTWNPAWTVSTIITGLMSFMND..NQ
Sp_Ubc6_5 59 YPFKPPAIRMIT...PSGRFQTNTRLCLSFSDFHP.........KSWNPSWMVSTILVGLVSFMTS..DE
At_Ubc33_5 60 YPYKPPGITMTT...PNGRFVTQKKICLSMSDFHP.........ESWNPMWSVSSILTGLLSFMMD..NS
At_Ubc34_5 60 YPYKPPGITMTT...PNGRFMTQKKICLSMSDFHP.........ESWNPMWSVSSILTGLLSFMMD..TS
Le_Ubc_5 60 YPFKPPGISMTT...PNGRFMTQKKICLSMSDFHP.........ESWNPMWSVSSILTGLLSFMMD..NS
α3 α4
Sc_Ubc3_3
Sc_Ubc3_3 138 NSPANVDAAVDYRKNPEQYKQRVKMEVERSKQD
Hs_UBE2J2_5 116 PTLGSIET..........SDFTKRQLAVQS...
Sc_Ubc6_5 115 ATTGSITT..........SDHQKKTLARNS...
Ce_Ubc15_5 116 PAAGSIAG..........TPQDQRMYAAAS...
Pm_Sb55_5 116 PTTGSVST..........TVADKQRLAKAS...
At_Ubc32_5 119 GALGSVDY..........PKDERRTLAIKS...
Hs_UBE2J1_5 116 GAIGSLDY..........TPEERRALAKKSQDF
Mm_Ube2J1_5 116 GAIGSLDY..........TPEERRALAKKSQDF
Mm_Ube2J2_5 116 PTLGSIET..........SDFTKKQLAAQSLVF
Dm_CG5823_5 116 PTLGSIES..........SNYDKQMFAQKSLAF
Ce_Ubc6_5 116 GALGSLDY..........PPKERQRLAKLSCEW
Ce_Ubc26_5 116 PTLGSLVT..........SESERKLLAKKSKQW
Sp_Ubc6_5 115 ITTGGIVT..........SESTRRTYAKDTKRF
At_Ubc33_5 116 PTTGSVNT..........SVAEKQRLAKSSLAF
At_Ubc34_5 116 PTTGSVNT..........TVIEKQRLAKSSLAF
Le_Ubc_5 116 PTTGSVST..........TVAEKKKLAKASLAF

```
                         α1                  β1           β2                 β3
Sc_Ubc3_3
Sc_Ubc3_3         1  .MSS..RKSTASSLLRQY.RELTDPKKAIPSFHIELEDDSNIFTWNIGVMVLNEDSIYHGGFFKAQMRFP
Hs_UBE2H_6        1  .MSSPS.PGKRR.MDTDVVKLIE.SKHEV..TILGG.....LNEFVVKFYGP.QGTPYEGGVWKVRVDLP
Ce_Ubc8_6         1  .MTSATAIGKRR.IDCDVVKLIS.HNHEV..QIVNG.....CSEFIVRFHGP.KDTAYENGVWRIRVDMP
At_Ubc6_6         1  .MASP...SKRR..EMDMMKLMM.SDYKV..DTVN..DD..LQMFYVTFHGP.TDSLYQGGVWKIKVELP
Sc_Ubc8_6         1  .MSS....SKRR.IETDVMKLLM.SDHQV..DLIN..DS..MQEFHVKFLGP.KDTPYENGVWRLHVELP
Sp_Ubc8_6         1  .MSSP....RRR.IETDVMKLLM.SDYEV..TLVN..DN..MQEFYVRFHGP.SETPYSGGIWKVHVELP
Dm_CG14739_6      1  LVNTTLPMAGRR.LDRDVNRLLA.SGYR...TTVD..DD..MTNLNVCLEGP.LGSAYEGGIWTVNVTMP
Mm_Ube2H_6        1  .MSSPS.PGKRR.MDTDVVKLIE.SKHEV..TILGG.....LNEFVVKFYGP.QGTPYEGGVWKVRVDLP
Dm_UbcDE2H_6      1  .MSSPS.AGKRR.MDNDVIKLIE.SKHEV..TILGG.....LNEFHVKFFGP.TETPYEGGVWKVRVYLP
At_Ubc5_6         1  .MSSP...SKRR..EMDLMKLMM.SDYKV..EMIN..DG..MQEFFVEFSGP.KDSIYEGGVWKIRVELP
At_Ubc4_6         1  .MSSP...SKRR..EMDMMKLMM.SDYKV..ETIN..DG..MQEFYVEFNGP.KDSLYQGGVWKIRVELP
Ta_Ubc4_6         1  .MSSP...SKRR..EMDLMKLMM.SDYKV..DMIN..DG..MHEFFVHFHGP.KDSIYQGGVWKVRVELT

                         β4                                                    α2
Sc_Ubc3_3
Sc_Ubc3_3        67  EDFPFSPPQFRFTPAIYHPNVY.RDGRLCISILHQSGDPMTDEPDAETWSPVQTVESVLISIVS.LLEDP
Hs_UBE2H_6       59  DKYPFKSPSIGFMNKIFHPNIDEASGTVCLDVINQ............TWTALYDLTNIFESFLPQLLAYP
Ce_Ubc8_6        60  DKYPFKSPSIGFLNKIFHPNIDEASGTVCLDVINQ............AWTALYDLTNIFDTFLPQLLTYP
At_Ubc6_6        57  EAYPYKSPSVGFVNKIYHPNVDESSGAVCLDVINQ............TWSPMFDLINVFESFLPQLLLYP
Sc_Ubc8_6        57  DNYPYKSPSIGFVNKIFHPNIDIASGSICLDVINS............TWSPLYDLINIVEWMIPGLLKEP
Sp_Ubc8_6        57  SEYPWKSPSIGFVNRIFHPNIDELSGSVCLDVINQ............TWSPMFDMINIFEVFLPQLLRYP
Dm_CG14739_6     61  QDYPLTAPRVRFVTKILHPNIEFITGLVCMNVLKQ............AWSSSYDLVNIFETFLPQLLRYP
Mm_Ube2H_6       59  DKYPFKSPSIGFMNKIFHPNIDEASGTVCLDVINQ............TWTALYDLTNIFESFLPQLLAYP
Dm_UbcDE2H_6     59  DNYPFKSPSIGFVNKIYHPNIDESSGTVCLDVINQ............AWTALYDLSNIFESFLPQLLTYP
At_Ubc5_6        57  DAYPYKSPSVGFITKIYHPNVDEMSGSVCLDVINQ............TWSPMFDLVNVFETFLPQLLLYP
At_Ubc4_6        57  DAYPYKSPSVGFITKIYHPNVDELSGSVCLDVINQ............TWSPMFDLVNVFETFLPQLLLYP
Ta_Ubc4_6        57  EAYPYKSPSIGFTNKIYHPNVDEMSGSVCLDVINQ............TWSPMFDLVNIFEVFLPQLLLYP

                             α3            α4
Sc_Ubc3_3
Sc_Ubc3_3       135  NINSPANVDAAVDYRKNPEQYKQRVKMEVERSKQD....
Hs_UBE2H_6      117  NPIDPLNGDAAAMYLHRPEEYKQKIKEYIQKYAT.....
Ce_Ubc8_6       118  NAADPLNGEAARLYIHKPEEYRRTCREYVMRFASEH...
At_Ubc6_6       115  NPSDPFNGEAASLLMRDRAAYELKVKEYCEKYAKP....
Sc_Ubc8_6       115  NGSDPLNNEAATLQLRDKKLYEEKIKEYIDKYATKE...
Sp_Ubc8_6       115  NASDPLNGEAALLLREPNTYYAKVRDYIARYANKEDAD
Dm_CG14739_6    119  NPHDSLNHRAAAIMKHSEQLFREHVILCMKTYAMPA...
Mm_Ube2H_6      117  NPIDPLNGDAAAMYLHRPEEYKQKIKEYIQKYATEEALK
Dm_UbcDE2H_6    117  NPVDPLNRDAAALYLHEPEEYHRKVADYVQRYATEDALR
At_Ubc5_6       115  NPSDPLNGEAAALMMRDRPTYEQRVKEYCEKYAKP....
At_Ubc4_6       115  NPSDPLNGEAAALMMRDRPAYEQRVKEYCEKYAKP....
Ta_Ubc4_6       115  NPSDPLNGEAASLMMRDKNAYENKVKEYCERYAKP....
```

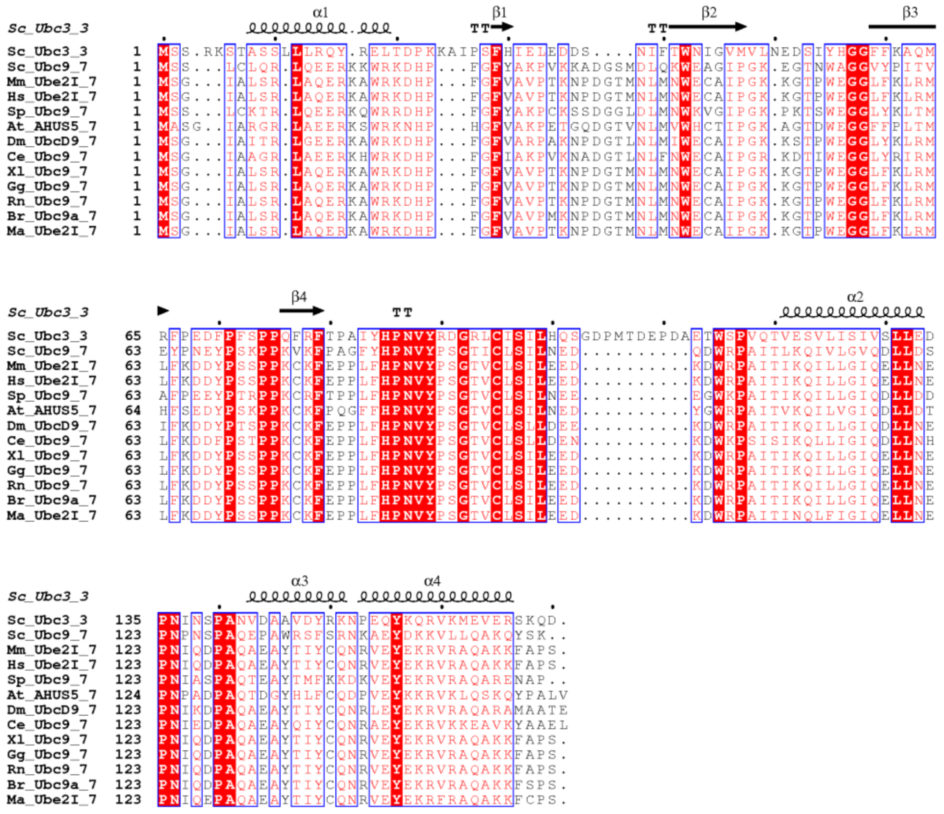

Sc_Ubc3_3
α1
β1
β2
β3
β4
α2
α3
α4
Sc_Ubc3_3
Sc_Ubc9_7
Mm_Ube2I_7
Hs_Ube2I_7
Sp_Ubc9_7
At_AHUS5_7
Dm_UbcD9_7
Ce_Ubc9_7
Xl_Ubc9_7
Gg_Ubc9_7
Rn_Ubc9_7
Br_Ubc9a_7
Ma_Ube2I_7

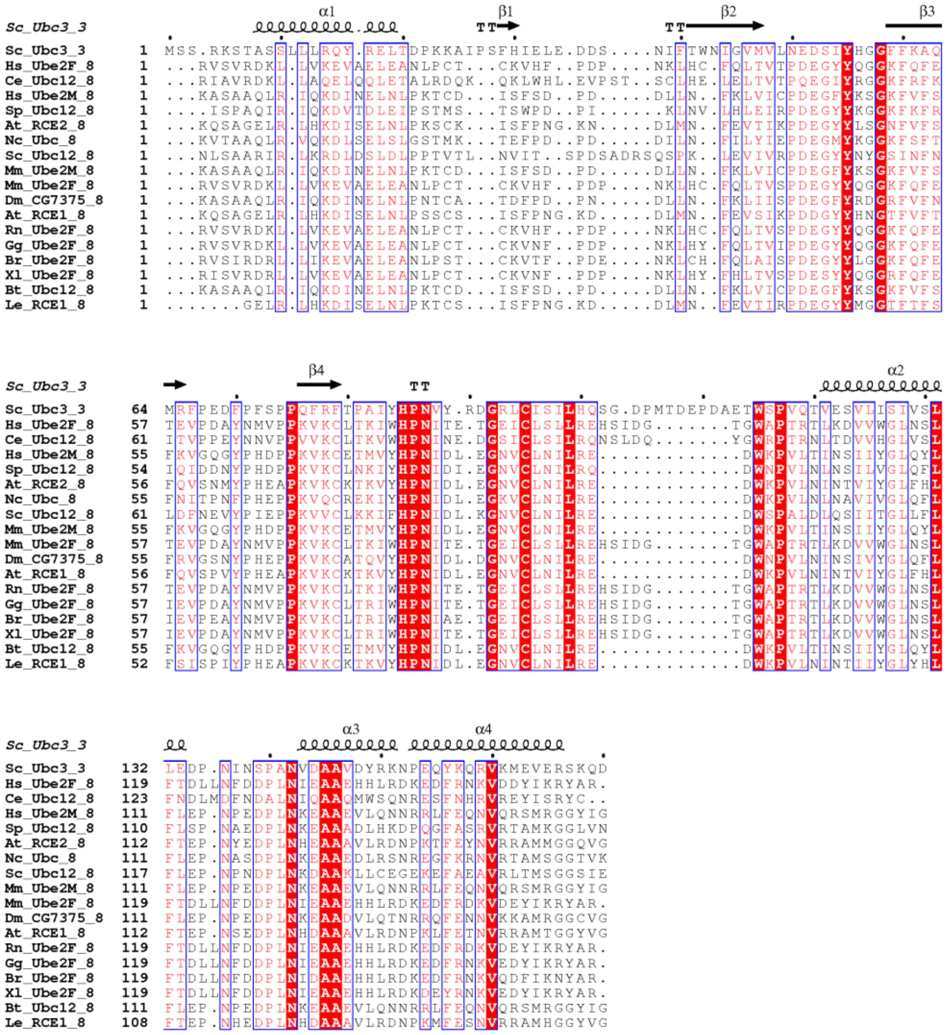

α1 β1 β2 β3
Sc_Ubc3_3
TT TT
Sc_Ubc3_3 1 MSS.RKSTASSLLLRQY.RELTDPKKAIPSFHIELE.DDS....NIFTWNIGVMVLNEDSIYHGGFFKAQ
Hs_Ube2F_8 1 ...RVSVRDKL.LVKEVAELEANLPCT...CKVHF..PDP....NKLHC.FQLTVTPDEGYYQGGKFQFE
Ce_Ubc12_8 1 ...RIAVRDKL.LAQELQQLETALRDQK..QKLWHL.EVPST..SCLHE.LELTVTPQEGIYRGGKFRFK
Hs_Ube2M_8 1 ...KASAAQLR.IQKDINELNLPKTCD...ISFSD..PD.....DLLN..FKLVICPDEGFYKSGKFVFS
Sp_Ubc12_8 1 ....ISPAQIR.IQKDVTDLEIPSTMS...TSWPD..PI......KLNV.LHLEIRPDEGYYKGGKFKFR
At_RCE2_8 1 ...KQSAGELR.LHKDISELNLPKSCK...ISFPNG.KN.....DLMN..FEVTIKPDEGYYLSGNFVFS
Nc_Ubc_8 1 ...KVTAAQLR.VQKDLSELSLGSTMK...TEFPD..PD.....DILN..FILYIEPDEGMYKGGKFSFT
Sc_Ubc12_8 1 ...NLSAARIR.LKRDLDSLDLPPTVTL..NVIT..SPDSADRSQSPK..LEVIVRPDEGYYNYGSINFN
Mm_Ube2M_8 1 ...KASAAQLR.IQKDINELNLPKTCD...ISFSD..PD.....DLLN..FKLVICPDEGFYKSGKFVFS
Mm_Ube2F_8 1 ...RVSVRDKL.LVKEVAELEANLPCT...CKVHF..PDP....NKLHC.FQLTVSPDEGYYQGGKFQFE
Dm_CG7375_8 1 ...KASAAQLR.IQKDINELNLPNTCA...TDFPD..PN.....DLLN..FKLIISPDEGFYRDGRFVFN
At_RCE1_8 1 ...KQSAGELR.LHKDISELNLPSSCS...ISFPNG.KD.....DLMN..FEVSIKPDDGYYHNGTFVFT
Rn_Ube2F_8 1 ...RVSVRDKL.LVKEVAELEANLPCT...CKVHF..PDP....NKLHC.FQLTVSPDEGYYQGGKFQFE
Gg_Ube2F_8 1 ...RVSVRDKL.LVKEVAELEANLPCT...CKVNF..PDP....NKLHY.FQLTVIPDEGYYQGGKFQFE
Br_Ube2F_8 1 ...RVSIRDRL.LIKEVAELEANLPST...CKVTF..PDE....NKLCH.FQLAISPDEGYYLGGKFQFE
Xl_Ube2F_8 1 ...RISVRDRL.LVKEVAELEANLPCT...CKVNF..PDP....NKLHY.FHLTVSPDESYYQGGRFQFE
Bt_Ubc12_8 1 ...KASAAQLR.IQKDINELNLPKTCD...ISFSD..PD.....DLLN..FKLVICPDEGFYKSGKFVFS
Le_RCE1_8 1 .......GELR.LHKDISELNLPKTCS...ISFPNG.KD.....DLMN..FEVTIRPDEGYYMGGTFTFS
β4 α2
Sc_Ubc3_3
TT
Sc_Ubc3_3 64 MRFPEDFPFSPPQFRFTPAIYHPNVY.RDGRLCISILHQSG.DPMTDEPDAETWSPVQTVESVLISIVSL
Hs_Ube2F_8 57 TEVPDAYNMVPPKVKCLTKIWHPNITE.TGEICLSLLREHSIDG.......TGWAPTRTLKDVVWGLNSL
Ce_Ubc12_8 61 ITVPPEYNNVPPVVKCLTKVWHPNINE.DGSICLSILRQNSLDQ.......YGWRPTRNLTDVVHGLVSL
Hs_Ube2M_8 55 FKVGQGYPHDPPKVKCETMVYHPNIDL.EGNVCLNILRE.............DWKPVLTINSIIYGLQYL
Sp_Ubc12_8 54 IQIDDNYPHDPPKVKCLNKIYHPNIDI.EGNVCLNILRQ.............DWNPVLNLNSILVGLQFL
At_RCE2_8 56 FQVSNMYPHEAPKVKCKTKVYHPNIDL.EGNVCLNILRE.............DWKPVLNINTVIYGLFHL
Nc_Ubc_8 55 FNITPNFPHEPPKVQCREKIYHPNIDL.EGKVCLNILRE.............DWKPVLNLNAVIVGLQFL
Sc_Ubc12_8 61 LDFNEVYPIEPPKVVCLKKIFHPNIDL.KGNVCLNILRE.............DWSPALDLQSIITGLLFL
Mm_Ube2M_8 55 FKVGQGYPHDPPKVKCETMVYHPNIDL.EGNVCLNILRE.............DWKPVLTINSIIYGLQYL
Mm_Ube2F_8 57 TEVPDAYNMVPPKVKCLTKIWHPNITE.TGEICLSLLREHSIDG.......TGWAPTRTLKDVVWGLNSL
Dm_CG7375_8 55 FRVGSNYPHEPPKVKCATQVYHPNIDL.DGNVCLNILRE.............DWNPVLNINSIVYGLQFL
At_RCE1_8 56 FQVSPVYPHEAPKVKCKTKVYHPNIDL.EGNVCLNILRE.............DWKPVLNINTVIYGLFHL
Rn_Ube2F_8 57 TEVPDAYNMVPPKVKCLTKIWHPNITE.TGEICLSLLREHSIDG.......TGWAPTRTLKDVVWGLNSL
Gg_Ube2F_8 57 IEVPDAYNMVPPKVKCLTRIWHPNITE.TGEICLSLLREHSIDG.......TGWAPTRTLKDVVWGLNSL
Br_Ube2F_8 57 IEVPEAYNMVPPKVKCLTRIWHPNIAE.TGEICLSLLREHSIDG.......TGWAPTRTLKDVVWGLNSL
Xl_Ube2F_8 57 IEVPDAYNMVPPKVKCLTRIWHPNITE.TGEICLSLLREHSIDG.......TGWAPTRTLKDVVWGLNSL
Bt_Ubc12_8 55 FKVGQGYPHDPPKVKCETMVYHPNIDL.EGNVCLNILRE.............DWKPVLTINSIIYGLQYL
Le_RCE1_8 52 FSISPIYPHEAPKVKCKTKVYHPNIDL.EGNVCLNILRE.............DWKPVLNINTIIYGLYHL
α3 α4
Sc_Ubc3_3
Sc_Ubc3_3 132 LEDP.NINSPANVDAAVDYRKNPEQYKQRVKMEVERSKQD
Hs_Ube2F_8 119 FTDLLNFDDPLNIEAAEHHLRDKEDFRNKVDDYIKRYAR.
Ce_Ubc12_8 123 FNDLMDFNDALNIQAAQMWSQNRESFNHRVREYISRYC..
Hs_Ube2M_8 111 FLEP.NPEDPLNKEAAEVLQNNRRLFEQNVQRSMRGGYIG
Sp_Ubc12_8 110 FLSP.NAEDPLNKEAAADLHKDPQGFASRVRTAMKGGLVN
At_RCE2_8 112 FTEP.NYEDPLNHEAAAVLRDNPKTFEYNVRRAMMGGQVG
Nc_Ubc_8 111 FLEP.NASDPLNKEAAEDLRSNREGFKRNVRTAMSGGTVK
Sc_Ubc12_8 117 FLEP.NPNDPLNKDAAKLLCEGEKEFAEAVRLTMSGGSIE
Mm_Ube2M_8 111 FLEP.NPEDPLNKEAAEVLQNNRRLFEQNVQRSMRGGYIG
Mm_Ube2F_8 119 FTDLLNFDDPLNIEAAEHHLRDKEDFRDKVDEYIKRYAR.
Dm_CG7375_8 111 FLEP.NPEDPLNKEAADVLQTNRRQFENNVKKAMRGGCVG
At_RCE1_8 112 FTEP.NSEDPLNHDAAAVLRDNPKLFETNVRRAMTGGYVG
Rn_Ube2F_8 119 FTDLLNFDDPLNIEAAEHHLRDKEDFRDKVDEYIKRYAR.
Gg_Ube2F_8 119 FTDLLNFDDPLNIEAAEHHLRDKEDFRNKVEDYIKRYAR.
Br_Ube2F_8 119 FTDLLNFDDPLNIDAAEHHLRDKEDFRNKVQDFIKNYAR.
Xl_Ube2F_8 119 FTDLLNFDDPLNIEAAEHHLRDKDEYRNKVEDYIKRYAR.
Bt_Ubc12_8 111 FLEP.NPEDPLNKEAAEVLQNNRRLFEQNVQRSMRGGYIG
Le_RCE1_8 108 FTEP.NHEDPLNHDAAAVLRDNPKMFESNVRRAMHGGYVG

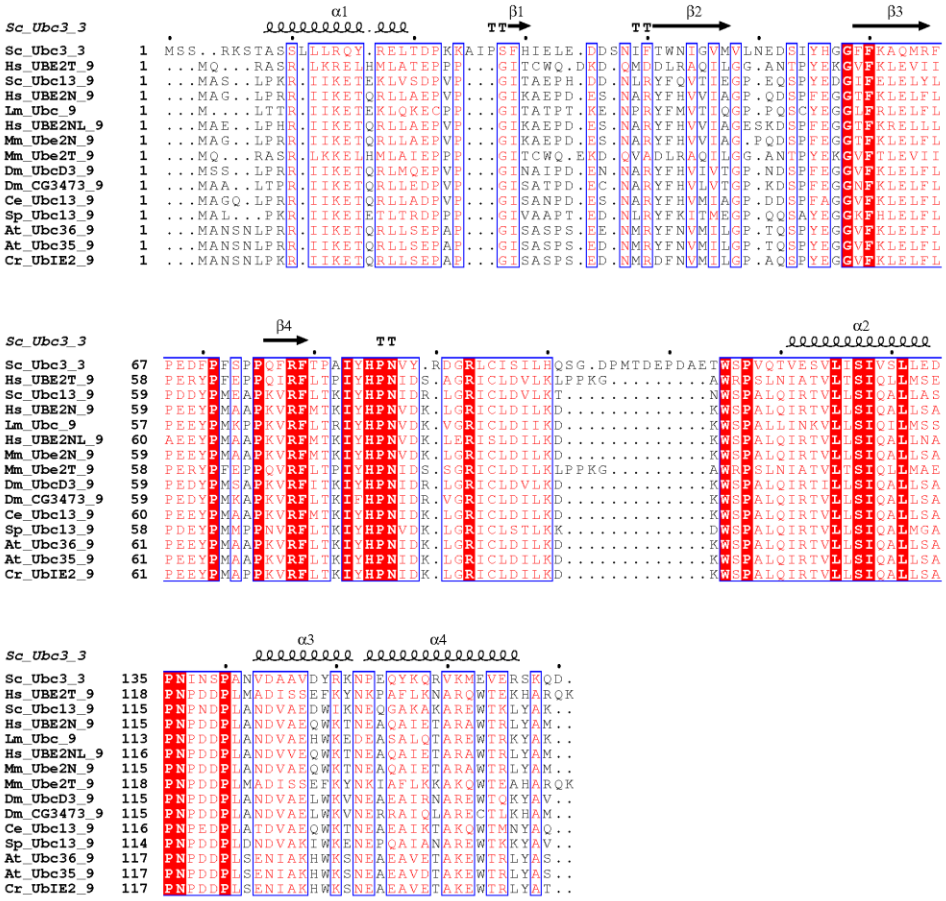

Sc_Ubc3_3
α1
β1
β2
β3
β4
α2
α3
α4
TT
Sc_Ubc3_3
Hs_UBE2T_9
Sc_Ubc13_9
Hs_UBE2N_9
Lm_Ubc_9
Hs_UBE2NL_9
Mm_Ube2N_9
Mm_Ube2T_9
Dm_UbcD3_9
Dm_CG3473_9
Ce_Ubc13_9
Sp_Ubc13_9
At_Ubc36_9
At_Ubc35_9
Cr_UbIE2_9

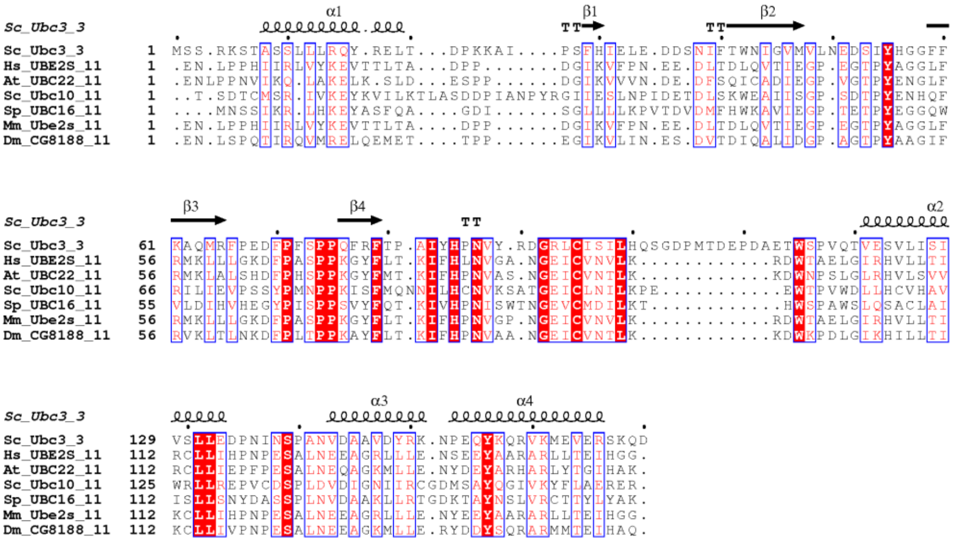
α1 β1 β2
Sc_Ubc3_3
Sc_Ubc3_3 1 MSS.RKSTASSLLLRQY.RELT...DPKKAI....PSFHIELE.DDSNIFTWNIGVMVLNEDSIYHGGFF
Hs_UBE2S_11 1 .EN.LPPHIIRLVYKEVTTLTA...DPP.......DGIKVFPN.EE.DLTDLQVTIEGP.EGTPYAGGLF
At_UBC22_11 1 .ENLPPNVIKQ.LAKELK.SLD...ESPP......DGIKVVVN.DE.DFSQICADIEGP.VGTPYENGLF
Sc_Ubc10_11 1 ..T.SDTCMSR.IVKEYKVILKTLASDDPIANPYRGIIESLNPIDETDLSKWEAIISGP.SDTPYENHQF
Sp_UBC16_11 1 ....MNSSIKR.LHKEYASFQA....GDI......SGLLLLKPVTDVDMFHWKAVIEGP.TETPYEGGQW
Mm_Ube2s_11 1 .EN.LPPHIIRLVYKEVTTLTA....DPP......DGIKVFPN.EE.DLTDLQVTIEGP.EGTPYAGGLF
Dm_CG8188_11 1 .EN.LSPQTIRQVMRELQEMET....TPP......EGIKVLIN.ES.DVTDIQALIDGP.AGTPYAAGIF
β3 β4 α2
Sc_Ubc3_3
Sc_Ubc3_3 61 KAQMRFPEDFPFSPPQFRFTP.AIYHPNVY.RDGRLCISILHQSGDPMTDEPDAETWSPVQTVESVLISI
Hs_UBE2S_11 56 RMKLLLGKDFPASPPKGYFLT.KIFHLNVGA.NGEICVNVLK............RDWTAELGIRHVLLTI
At_UBC22_11 56 RMKLALSHDFPHSPPKGYFMT.KIFHPNVAS.NGEICVNTLK............KDWNPSLGLRHVLSVV
Sc_Ubc10_11 66 RILIEVPSSYPMNPPKISFMQNNILHCNVKSATGEICLNILKPE...........EWTPVWDLLHCVHAV
Sp_UBC16_11 55 VLDIHVHEGYPISPPSVYFQT.KIVHPNISWTNGEVCMDILKT............HWSPAWSLQSACLAI
Mm_Ube2s_11 56 RMKLLLGKDFPASPPKGYFLT.KIFHPNVGP.NGEICVNVLK............RDWTAELGIRHVLLTI
Dm_CG8188_11 56 RVKLTLNKDFPLTPPKAYFLT.KIFHPNVAA.NGEICVNTLK............KDWKPDLGIKHILLTI
α3 α4
Sc_Ubc3_3
Sc_Ubc3_3 129 VSLLEDPNINSPANVDAAVDYRK.NPEQYKQRVKMEVERSKQD
Hs_UBE2S_11 112 RCLLIHPNPESALNEEAGRLLLE.NSEEYAARARLLTEIHGG.
At_UBC22_11 112 RCLLIEPFPESALNEQAGKMLLE.NYDEYARHARLYTGIHAK.
Sc_Ubc10_11 125 WRLLREPVCDSPLDVDIGNIIRCGDMSAYQGIVKYFLAERER.
Sp_UBC16_11 112 ISLLSNYDASSPLNVDAAKLLRTGDKTAYNSLVRCTTYLYAK.
Mm_Ube2s_11 112 KCLLIHPNPESALNEEAGRLLLE.NYEEYAARARLLTEIHGG.
Dm_CG8188_11 112 KCLLIVPNPESALNEEAGKMLLE.RYDDYSQRARMMTEIHAQ.

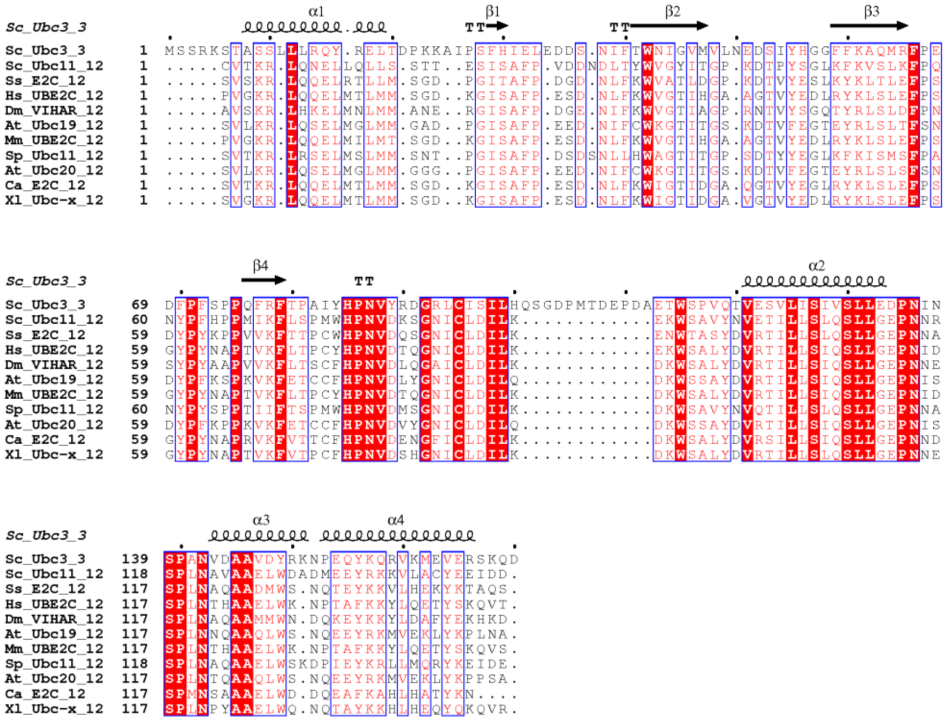
Sc_Ubc3_3
α1
β1
β2
β3
TT
TT
Sc_Ubc3_3 1 MSSRKSTASSLLLRQY.RELTDPKKAIPSFHIELEDDS.NIFTWNIGVMVLNEDSIYHGGFFKAQMRFPE
Sc_Ubc11_12 1 .....CVTKR.LQNELLQLLS.STT..ESISAFP.VDDNDLTYWVGYITGP.KDTPYSGLKFKVSLKFPQ
Ss_E2C_12 1 .....SVSKR.LQQELRTLLM.SGD..PGITAFP.DGD.NLFKWVATLDGP.KDTVYESLKYKLTLEFPS
Hs_UBE2C_12 1 .....PVGKR.LQQELMTLMM.SGD..KGISAFP.ESD.NLFKWVGTIHGA.AGTVYEDLRYKLSLEFPS
Dm_VIHAR_12 1 .....AVSKR.LHKELMNLMM.ANE..RGISAFP.DGE.NIFKWVGTIAGP.RNTVYSGQTYRLSLDFPN
At_Ubc19_12 1 .....SVLKR.LQSELMGLMM.GAD..PGISAFP.EED.NIFCWKGTITGS.KDTVFEGTEYRLSLTFSN
Mm_UBE2C_12 1 .....PVGKR.LQQELMILMT.SGD..KGISAFP.ESD.NLFKWVGTIHGA.AGTVYEDLRYKLSLEFPS
Sp_Ubc11_12 1 .....SVTKR.LRSELMSLMM.SNT..PGISAFP.DSDSNLLHWAGTITGP.SDTYYEGLKFKISMSFPA
At_Ubc20_12 1 .....SVLKR.LQSELMGLMM.GGG..PGISAFP.EED.NIFCWKGTITGS.KDTVFEGTEYRLSLSFSN
Ca_E2C_12 1 .....SVTKR.LQQELMTLMM.SGD..KGISAFP.ESD.NLFKWIGTIDGA.QGTVYEGLRYKLSLEFPS
Xl_Ubc-x_12 1 .....SVGKR.LQQELMTLMM.SGD..KGISAFP.ESD.NLFKWIGTIDGA.VGTVYEDLRYKLSLEFPS
Sc_Ubc3_3
β4
TT
α2
Sc_Ubc3_3 69 DFPFSFPQFRFTPAIYHPNVYRDGRLCISILHQSGDPMTDEPDAETWSPVQTVESVLISIVSLLEDPNIN
Sc_Ubc11_12 60 NYPFHPPMIKFLSPMWHPNVDKSGNICLDILK............EKWSAVYNVETILLSLQSLLGEPNNR
Ss_E2C_12 59 DYPYKPPVVKFTTPCWHPNVDQSGNICLDILK............ENWTASYDVRTILLSLQSLLGEPNNA
Hs_UBE2C_12 59 GYPYNAPTVKFLTPCYHPNVDTQGNICLDILK............EKWSALYDVRTILLSIQSLLGEPNID
Dm_VIHAR_12 59 SYPYAAPVVKFLTSCFHPNVDLQGAICLDILK............DKWSALYDVRTILLSIQSLLGEPNNE
At_Ubc19_12 59 DYPFKSPKVKFETCCFHPNVDLYGNICLDILQ............DKWSSAYDVRTILLSIQSLLGEPNIS
Mm_UBE2C_12 59 GYPYNAPTVKFLTPCYHPNVDTQGNICLDILK............DKWSALYDVRTILLSIQSLLGEPNID
Sp_Ubc11_12 60 NYPYSFPTIIFTSPMWHPNVDMSGNICLDILK............DKWSAVYNVQTILLSLQSLLGEPNNA
At_Ubc20_12 59 DYPFKPPKVKFETCCFHPNVDVYGNICLDILQ............DKWSSAYDVRTILLSIQSLLGEPNIS
Ca_E2C_12 59 GYPYNAPRVKFVTTCFHPNVDENGFICLDILK............DKWSALYDVRSILLSIQSLLGEPNND
Xl_Ubc-x_12 59 GYPYNAPTVKFVTPCFHPNVDSHGNICLDILK............DKWSALYDVRTILLSIQSLLGEPNNE
Sc_Ubc3_3
α3
α4
Sc_Ubc3_3 139 SPANVDAAVDYRKNPEQYKQRVKMEVERSKQD
Sc_Ubc11_12 118 SPLNAVAAELWDADMEEYRKKVLACYEEIDD.
Ss_E2C_12 117 SPLNAQAADMWS.NQTEYKKVLHEKYKTAQS.
Hs_UBE2C_12 117 SPLNTHAAELWK.NPTAFKKYLQETYSKQVT.
Dm_VIHAR_12 117 SPLNAQAAMMWN.DQKEYKKYLDAFYEKHKD.
At_Ubc19_12 117 SPLNNQAAQLWS.NQEEYRKMVEKLYKPLNA.
Mm_UBE2C_12 117 SPLNTHAAELWK.NPTAFKKYLQETYSKQVS.
Sp_Ubc11_12 118 SPLNAQAAELWSKDPIEYKRLLMQRYKEIDE.
At_Ubc20_12 117 SPLNTQAAQLWS.NQEEYRKMVEKLYKPPSA.
Ca_E2C_12 117 SPMNSAAAELWD.DQEAFKAHLHATYKN....
Xl_Ubc-x_12 117 SPLNPYAAELWQ.NQTAYKKHLHEQYQKQVR.

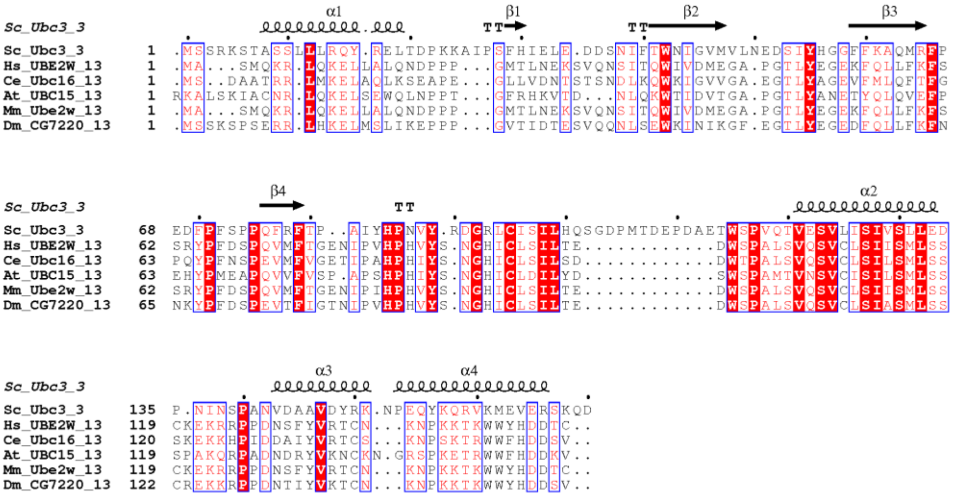
α1
β1
β2
β3
TT
TT
Sc_Ubc3_3
Sc_Ubc3_3 1 .MSSRKSTASSLLLRQY.RELTDPKKAIPSFHIELE.DDSNIFTWNIGVMVLNEDSIYHGGFFKAQMRFP
Hs_UBE2W_13 1 .MA...SMQKR.LQKELLALQNDPPP...GMTLNEKSVQNSITQWIVDMEGA.PGTLYEGEKFQLLFKFS
Ce_Ubc16_13 1 .MS..DAATRR.LMKELAQLKSEAPE...GLLVDNTSTSNDLKQWKIGVVGA.EGTLYAGEVFMLQFTFG
At_UBC15_13 1 RKALSKIACNR.LQKELSEWQLNPPT...GFRHKVTD...NLQKWTIDVTGA.PGTLYANETYQLQVEFP
Mm_Ube2w_13 1 .MA...SMQKR.LQKELLALQNDPPP...GMTLNEKSVQNSITQWIVDMEGA.PGTLYEGEKFQLLFKFS
Dm_CG7220_13 1 .MSSKSPSERR.LHKELMSLIKEPPP...GVTIDTESVQQNLSEWKINIKGF.EGTLYEGEDFQLLFKFN
β4
TT
α2
Sc_Ubc3_3
Sc_Ubc3_3 68 EDFPFSPPQFRFTP..AIYHPNVY.RDGRLCISILHQSGDPMTDEPDAETWSPVQTVESVLISIVSLLED
Hs_UBE2W_13 62 SRYPFDSPQVMFTGENIPVHPHVYS.NGHICLSILTE............DWSPALSVQSVCLSIISMLSS
Ce_Ubc16_13 63 PQYPFNSPEVMFVGETIPAHPHIYS.NGHICLSILSD............DWTPALSVQSVCLSILSMLSS
At_UBC15_13 63 EHYPMEAPQVVFVSP.APSHPHIYS.NGHICLDILYD............SWSPAMTVNSVCISILSMLSS
Mm_Ube2w_13 62 SRYPFDSPQVMFTGENIPIHPHVYS.NGHICLSILTE............DWSPALSVQSVCLSIISMLSS
Dm_CG7220_13 65 NKYPFDSPEVTFIGTNIPVHPHVYS.NGHICLSILTE............DWSPALSVQSVCLSIASMLSS
α3
α4
Sc_Ubc3_3
Sc_Ubc3_3 135 P.NINSPANVDAAVDYRK.NPEQYKQRVKMEVERSKQD
Hs_UBE2W_13 119 CKEKRRPPDNSFYVRTCN...KNPKKTKWWYHDDTC..
Ce_Ubc16_13 120 SKEKKHPIDDAIYVRTCS...KNPSKTRWWFHDDSV..
At_UBC15_13 119 SPAKQRPADNDRYVKNCKN.GRSPKETRWWFHDDKV..
Mm_Ube2w_13 119 CKEKRRPPDNSFYVRTCN...KNPKKTKWWYHDDTC..
Dm_CG7220_13 122 CREKKRPPDNTIYVKTCN...KNPKKTKWWYHDDSV..

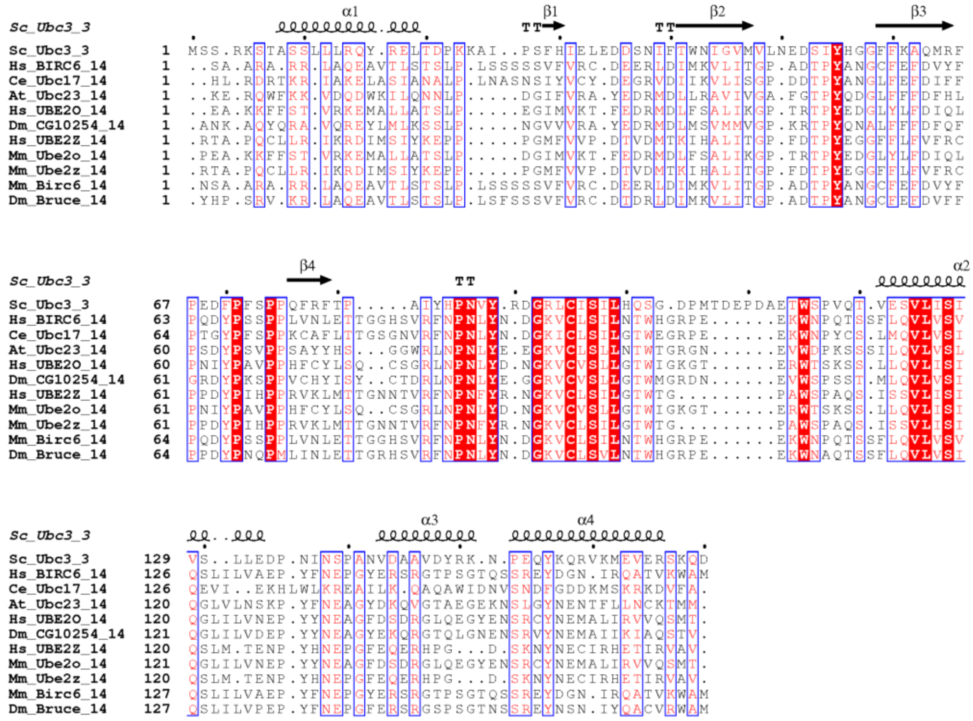
α1
β1
β2
β3
Sc_Ubc3_3
TT
Sc_Ubc3_3 1 MSS.RKSTASSLLLRQY.RELTDPKKAI..PSFHIELEDDSNIFTWNIGVMVLNEDSIYHGGFFKAQMRF
Hs_BIRC6_14 1 ..SA.ARA.RR.LAQEAVTLSTSLP.LSSSSSVFVRC.DEERLDIMKVLITGP.ADTPYANGCFEFDVYF
Ce_Ubc17_14 1 ..HL.RDRTKR.IAKELASIANALP.LNASNSIYVCY.DEGRVDIIKVLISGP.DDTPYANGLFEFDIFF
At_Ubc23_14 1 ..KE.RQWFKK.VDQDWKILQNNLP.....DGIFVRA.YEDRMDLLRAVIVGA.FGTPYQDGLFFFDFHL
Hs_UBE2O_14 1 ..EA.KKFFST.VRKEMALLATSLP.....EGIMVKT.FEDRMDLFSALIKGP.TRTPYEDGLYLFDIQL
Dm_CG10254_14 1 .ANK.AQYQRA.VQREYLMLKSSLP.....NGVVVRA.YEDRMDLMSVMMVGP.KRTPYQNALFFFDFQF
Hs_UBE2Z_14 1 .RTA.PQCLLR.IKRDIMSIYKEPP.....PGMFVVP.DTVDMTKIHALITGP.FDTPYEGGFFLFVFRC
Mm_Ube2o_14 1 .PEA.KKFFST.VRKEMALLATSLP.....DGIMVKT.FEDRMDLFSALIKGP.TRTPYEDGLYLFDIQL
Mm_Ube2z_14 1 .RTA.PQCLLR.IKRDIMSIYKEPP.....PGMFVVP.DTVDMTKIHALITGP.FDTPYEGGFFLFVFRC
Mm_Birc6_14 1 .NSA.ARA.RR.LAQEAVTLSTSLP.LSSSSSVFVRC.DEERLDIMKVLITGP.ADTPYANGCFEFDVYF
Dm_Bruce_14 1 .YHP.SRV.KR.LAQEAVTLSTSLP.LSFSSSVFVRC.DTDRLDIMKVLITGP.ADTPYANGCFEFDVFF
β4
α2
Sc_Ubc3_3
TT
Sc_Ubc3_3 67 PEDFPFSPPQFRFTP.....AIYHPNVY.RDGRLCISILHQSG.DPMTDEPDAETWSPVQT.VESVLISI
Hs_BIRC6_14 63 PQDYPSSPPLVNLETTGGHSVRFNPNLYN.DGKVCLSILNTWHGRPE......EKWNPQTSSFLQVLVSV
Ce_Ubc17_14 64 PTGYPFSPPKCAFLTTGSGNVRFNPNLYN.DGKICLSILGTWEGRPE......EKWNPYCS.LMQVLVSI
At_Ubc23_14 60 PSDYPSVPPSAYYHS...GGWRLNPNLYE.EGKVCLSLLNTWTGRGN......EVWDPKSSSILQVLVSL
Hs_UBE2O_14 60 PNIYPAVPPHFCYLSQ..CSGRLNPNLYD.NGKVCVSLLGTWIGKGT......ERWTSKSS.LLQVLISI
Dm_CG10254_14 61 GRDYPKSPPVCHYISY..CTDRLNPNLYE.GGRVCVSLLGTWMGRDN......EVWSPSST.MLQVLVSI
Hs_UBE2Z_14 61 PPDYPIHPPRVKLMTTGNNTVRFNPNFYR.NGKVCLSILGTWTG.........PAWSPAQS.ISSVLISI
Mm_Ube2o_14 61 PNIYPAVPPHFCYLSQ..CSGRLNPNLYD.NGKVCVSLLGTWIGKGT......ERWTSKSS.LLQVLISI
Mm_Ube2z_14 61 PPDYPIHPPRVKLMTTGNNTVRFNPNFYR.NGKVCLSILGTWTG.........PAWSPAQS.ISSVLISI
Mm_Birc6_14 64 PQDYPSSPPLVNLETTGGHSVRFNPNLYN.DGKVCLSILNTWHGRPE......EKWNPQTSSFLQVLVSV
Dm_Bruce_14 64 PPDYPNQPMLINLETTGRHSVRFNPNLYN.DGKVCLSVLNTWHGRPE......EKWNAQTSSFLQVLVSI
α3
α4
Sc_Ubc3_3
Sc_Ubc3_3 129 VS..LLEDP.NINSPANVDAAVDYRK.N.PEQYKQRVKMEVERSKQD
Hs_BIRC6_14 126 QSLILVAEP.YFNEPGYERSRGTPSGTQSSREYDGN.IRQATVKWAM
Ce_Ubc17_14 126 QEVI..EKHLWLKREAILK.QAQAWIDNVSNDFGDDKMSKRKDVFA.
At_Ubc23_14 120 QGLVLNSKP.YFNEAGYDKQVGTAEGEKNSLGYNENTFLLNCKTMM.
Hs_UBE2O_14 120 QGLILVNEP.YYNEAGFDSDRGLQEGYENSRCYNEMALIRVVQSMT.
Dm_CG10254_14 121 QGLILVDEP.YYNEAGYEKQRGTQLGNENSRVYNEMAIIKIAQSTV.
Hs_UBE2Z_14 120 QSLM.TENP.YHNEPGFEQERHPG...D.SKNYNECIRHETIRVAV.
Mm_Ube2o_14 121 QGLILVNEP.YYNEAGFDSDRGLQEGYENSRCYNEMALIRVVQSMT.
Mm_Ube2z_14 120 QSLM.TENP.YHNEPGFEQERHPG...D.SKNYNECIRHETIRVAV.
Mm_Birc6_14 127 QSLILVAEP.YFNEPGYERSRGTPSGTQSSREYDGN.IRQATVKWAM
Dm_Bruce_14 127 QSLILVPEP.YFNEPGFERSRGSPSGTNSSREYNSN.IYQACVRWAM

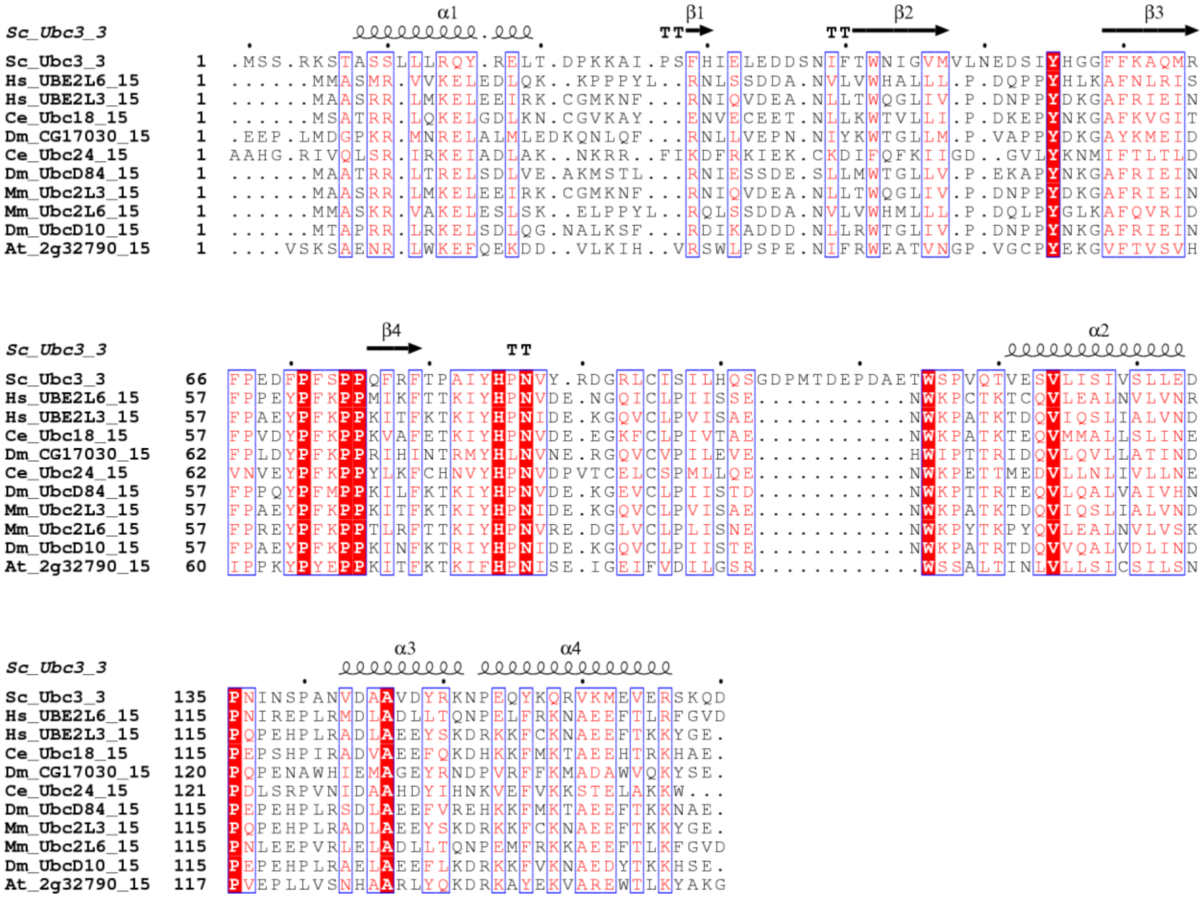
Sc_Ubc3_3
α1 β1 TT β2 TT β3
Sc_Ubc3_3 1 .MSS.RKSTASSLLLRQY.RELT.DPKKAI.PSFHIELEDDSNIFTWNIGVMVLNEDSIYHGGFFKAQMR
Hs_UBE2L6_15 1 ......MMASMR.VVKELEDLQK..KPPPYL..RNLSSDDA.NVLVWHALLL.P.DQPPYHLKAFNLRIS
Hs_UBE2L3_15 1 ......MAASRR.LMKELEEIRK.CGMKNF...RNIQVDEA.NLLTWQGLIV.P.DNPPYDKGAFRIEIN
Ce_Ubc18_15 1 ......MSATRR.LQKELGDLKN.CGVKAY...ENVECEET.NLLKWTVLLI.P.DKEPYNKGAFKVGIT
Dm_CG17030_15 1 .EEP.LMDGPKR.MNRELALMLEDKQNLQF...RNLLVEPN.NIYKWTGLLM.P.VAPPYDKGAYKMEID
Ce_Ubc24_15 1 AAHG.RIVQLSR.IRKEIADLAK..NKRR..FIKDFRKIEK.CKDIFQFKIIGD..GVLYKNMIFTLTLD
Dm_UbcD84_15 1 ......MAATRR.LTRELSDLVE.AKMSTL...RNIESSDE.SLLMWTGLLV.P.EKAPYNKGAFRIEIN
Mm_Ubc2L3_15 1 ......MAASRR.LMKELEEIRK.CGMKNF...RNIQVDEA.NLLTWQGLIV.P.DNPPYDKGAFRIEIN
Mm_Ubc2L6_15 1 ......MMASKR.VAKELESLSK..ELPPYL..RQLSSDDA.NVLVWHMLLL.P.DQLPYGLKAFQVRID
Dm_UbcD10_15 1 ......MTAPRR.LRKELSDLQG.NALKSF...RDIKADDD.NLLRWTGLIV.P.DNPPYNKGAFRIEIN
At_2g32790_15 1 ....VSKSAENR.LWKEFQEKDD..VLKIH..VRSWLPSPE.NIFRWEATVNGP.VGCPYEKGVFTVSVH
Sc_Ubc3_3
β4 TT α2
Sc_Ubc3_3 66 FPEDFPFSPPQFRFTPAIYHPNVY.RDGRLCISILHQSGDPMTDEPDAETWSPVQTVESVLISIVSLLED
Hs_UBE2L6_15 57 FPPEYPFKPPMIKFTTKIYHPNVDE.NGQICLPIISSE...........NWKPCTKTCQVLEALNVLVNR
Hs_UBE2L3_15 57 FPAEYPFKPPKITFKTKIYHPNIDE.KGQVCLPVISAE...........NWKPATKTDQVIQSLIALVND
Ce_Ubc18_15 57 FPVDYPFKPPKVAFETKIYHPNVDE.EGKFCLPIVTAE...........NWKPATKTEQVMMALLSLINE
Dm_CG17030_15 62 FPLDYPFKPPRIHINTRMYHLNVNE.RGQVCVPILEVE...........HWIPTTRIDQVLQVLLATIND
Ce_Ubc24_15 62 VNVEYPFKPPYLKFCHNVYHPNVDPVTCELCSPMLLQE...........NWKPETTMEDVLLNLIVLLNE
Dm_UbcD84_15 57 FPPQYPFMPPKILFKTKIYHPNVDE.KGEVCLPIISTD...........NWKPTTRTEQVLQALVAIVHN
Mm_Ubc2L3_15 57 FPAEYPFKPPKITFKTKIYHPNIDE.KGQVCLPVISAE...........NWKPATKTDQVIQSLIALVND
Mm_Ubc2L6_15 57 FPREYPFKPPTLRFTTKIYHPNVRE.DGLVCLPLISNE...........NWKPYTKPYQVLEALNVLVSK
Dm_UbcD10_15 57 FPAEYPFKPPKINFKTRIYHPNIDE.KGQVCLPIISTE...........NWKPATRTDQVVQALVDLIND
At_2g32790_15 60 IPPKYPYEPPKITFKTKIFHPNISE.IGEIFVDILGSR............WSSALTINLVLLSICSILSN
Sc_Ubc3_3
α3 α4
Sc_Ubc3_3 135 PNINSPANVDAAVDYRKNPEQYKQRVKMEVERSKQD
Hs_UBE2L6_15 115 PNIREPLRMDIADLLTQNPELFRKNAEEFTLRFGVD
Hs_UBE2L3_15 115 PQPEHPLRADLAEEYSKDRKKFCKNAEEFTKKYGE.
Ce_Ubc18_15 115 PEPSHPIRADVAEEFQKDHKKFMKTAEEHTRKHAE.
Dm_CG17030_15 120 PQPENAWHIEMAGEYRNDPVRFFKMADAWVQKYSE.
Ce_Ubc24_15 121 PDLSRPVNIDAAHDYIHNKVEFVKKSTELAKKW...
Dm_UbcD84_15 115 PEPEHPLRSDLAEEFVREHKKFMKTAEEFTKKNAE.
Mm_Ubc2L3_15 115 PQPEHPLRADLAEEYSKDRKKFCKNAEEFTKKYGE.
Mm_Ubc2L6_15 115 PNLEEPVRLELADLLTQNPEMFRKKAEEFTLKFGVD
Dm_UbcD10_15 115 PEPEHPLRAELAEEFLKDRKKFVKNAEDYTKKHSE.
At_2g32790_15 117 PVEPLLVSNHAARLYQKDRKAYEKVAREWTLKYAKG

Figure 2. Conformational landscape explored in Cdc34^{UBC} simulations. The free energy landscape is represented using projection of the Cdc34^{UBC} macro-trajectory along the principal components PC1 and PC2 of the essential subspace. The free energy is given in KJ/mol and indicated by the color bar. The label A-E indicates the region corresponding to the minimum free energy basins and the most populated structural clusters from cluster analysis.

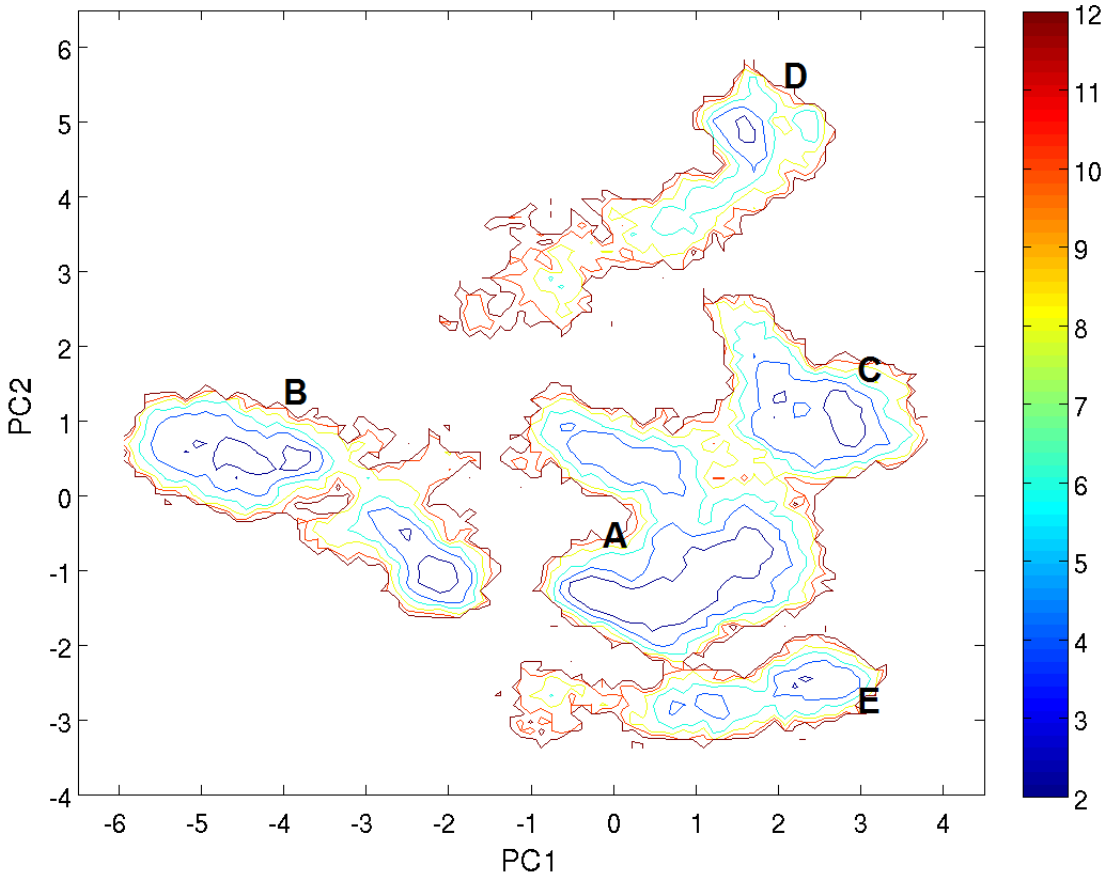

Figure 3. Flexibility profiles of non-phosphorylated Cdc34^{UBC}. Cα rmsf of ensemble trajectories of Cdc34^{UBC} and Cdc34^{UBC}–S130AS167A concatenated trajectories. The most persistent secondary structure during the simulations are represented schematically for each protein system. The rectangular box indicates the acidic loop.

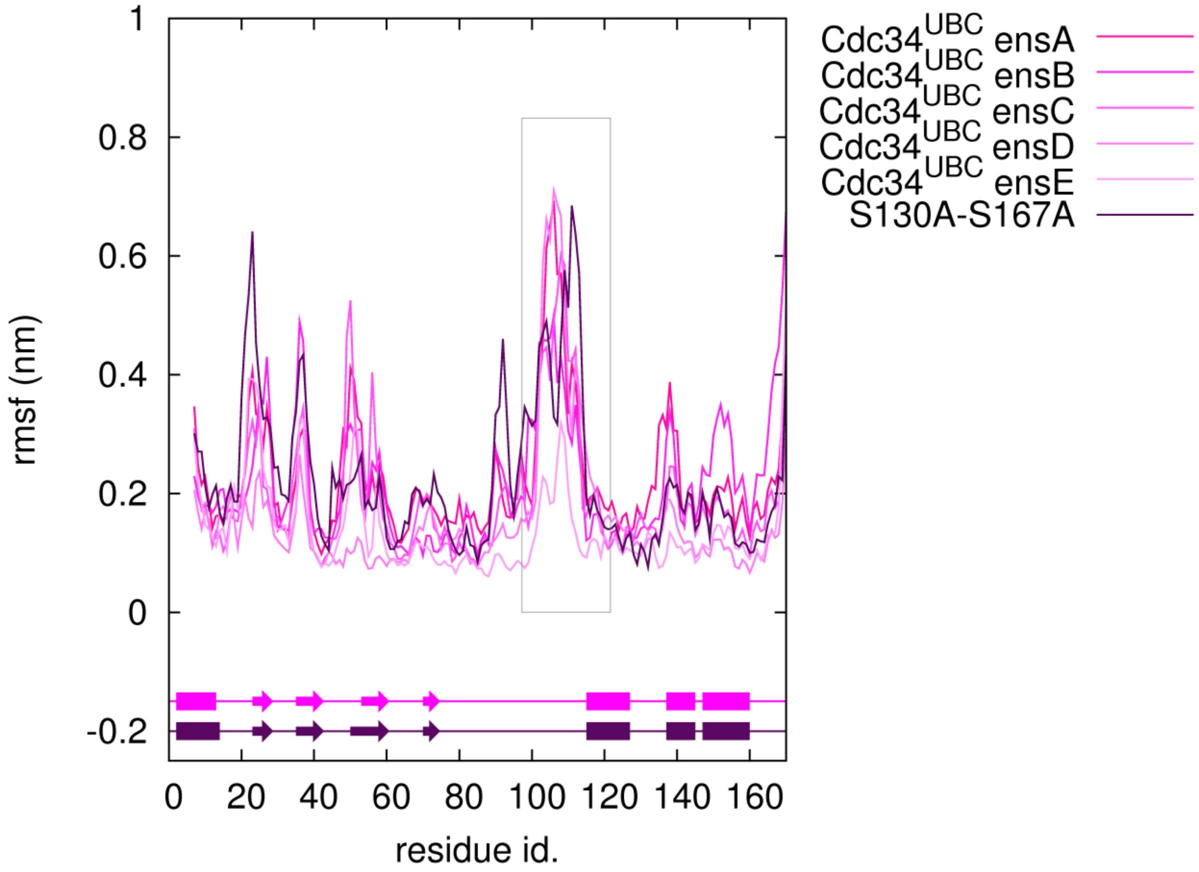

Figure 4. Flexibility profiles of phospho-Cdc34^{UBC}–pS130pS167 simulations. Cα rmsf of ensemble trajectories of Cdc34^{UBC}-pS130-pS167 and non-phosphorylated Cdc34^{UBC} are compared. The most persistent secondary structure during the simulations are represented schematically for each protein system. The rectangular box indicates the acidic loop.

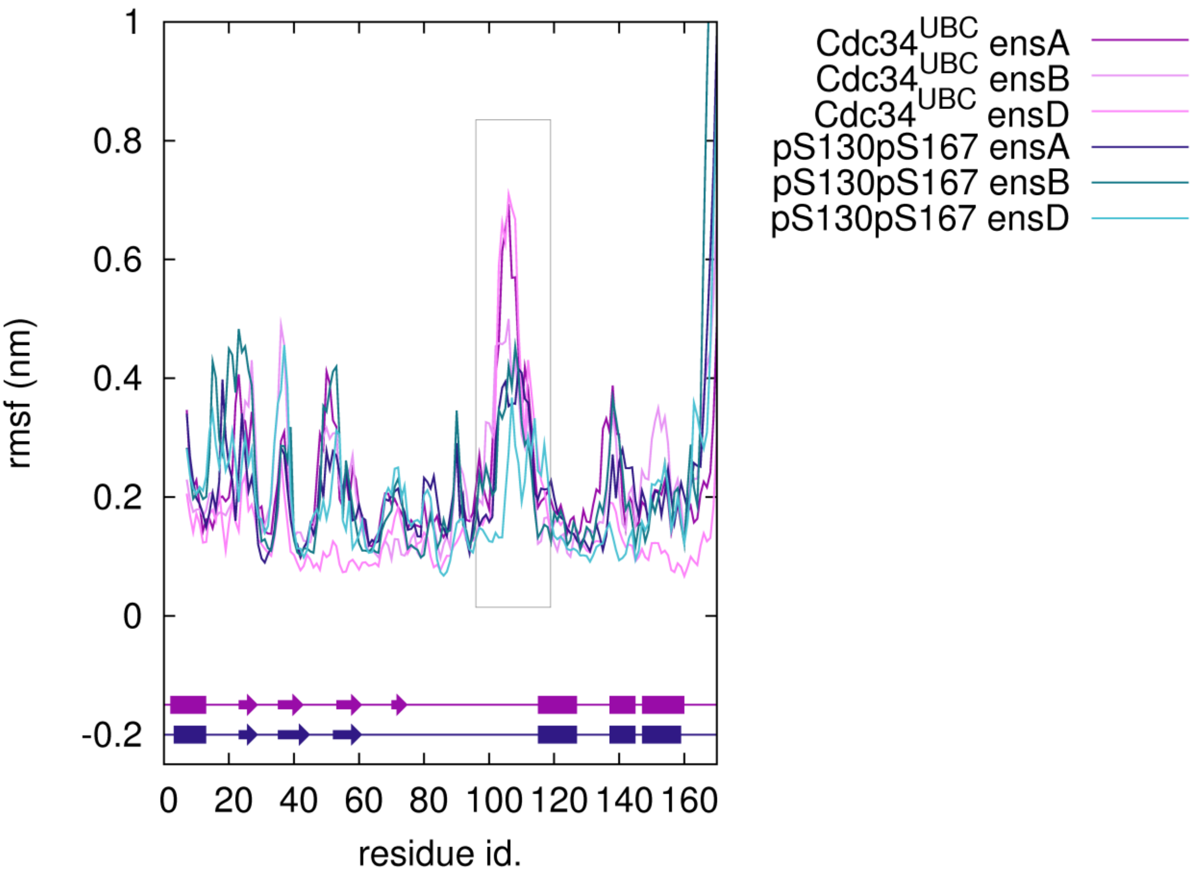

Figure 5. 3D structural superimposition of an average structure from phospho-Cdc34^{UBC}simulations, free NMR and X-ray structure of Ube2g2 and Ube2g2 in complex with the gp78 region of its E3 partner. The average structure of phospho Cdc34^{UBC} simulations is shown in blue, the NMR (PDB entry 2KLY) and X-ray (PDB entry 2CYX) structure of Ube2g2 are shown in dark and light green, respectively, the structure of Ube2g2 in complex with gp78 region of E3 partner (PDB entry 3H8K) is shown in orange. The catalytic cysteine is shown as yellow stick.

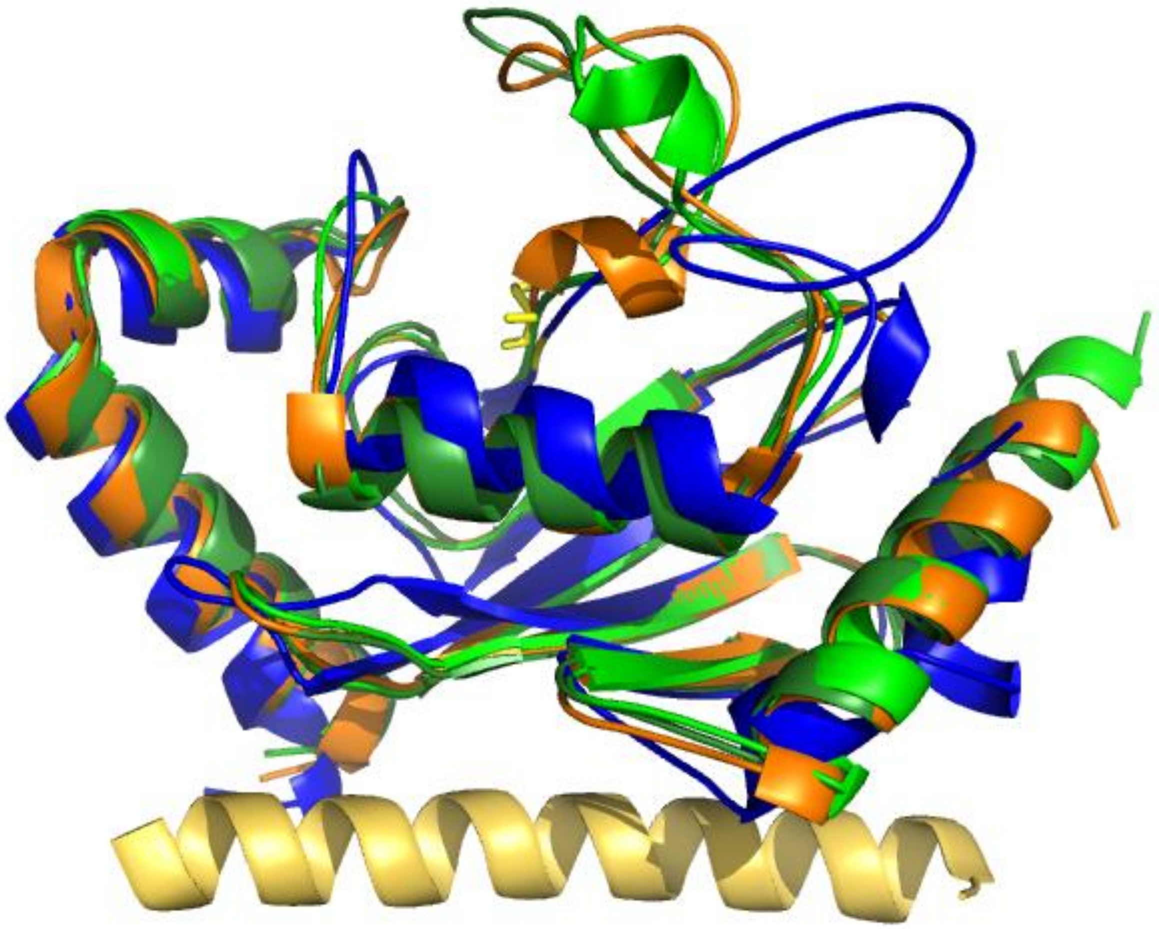

Figure 6. Projection of the simulations frames along the PC1 of Cdc34^{UBC}-S130D concatenated trajectory, indicated with different shades of grey. The rectangular box indicates the acidic loop and its aminoacidic composition.

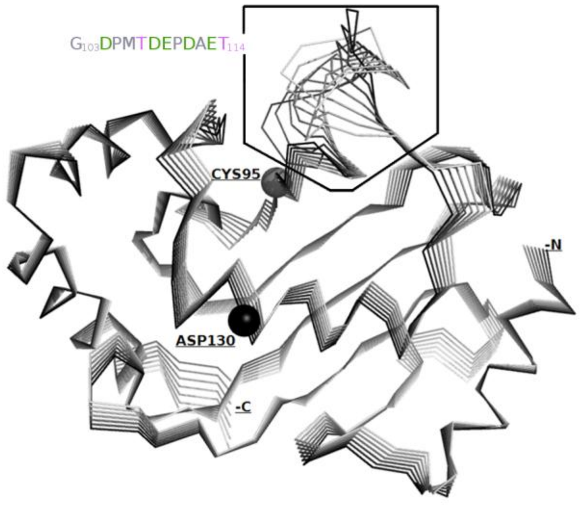

Figure 7. Root mean square fluctuation (rmsf) profiles of Cdc34^{UBC} and Cdc34-Δ12^{UBC} domains.

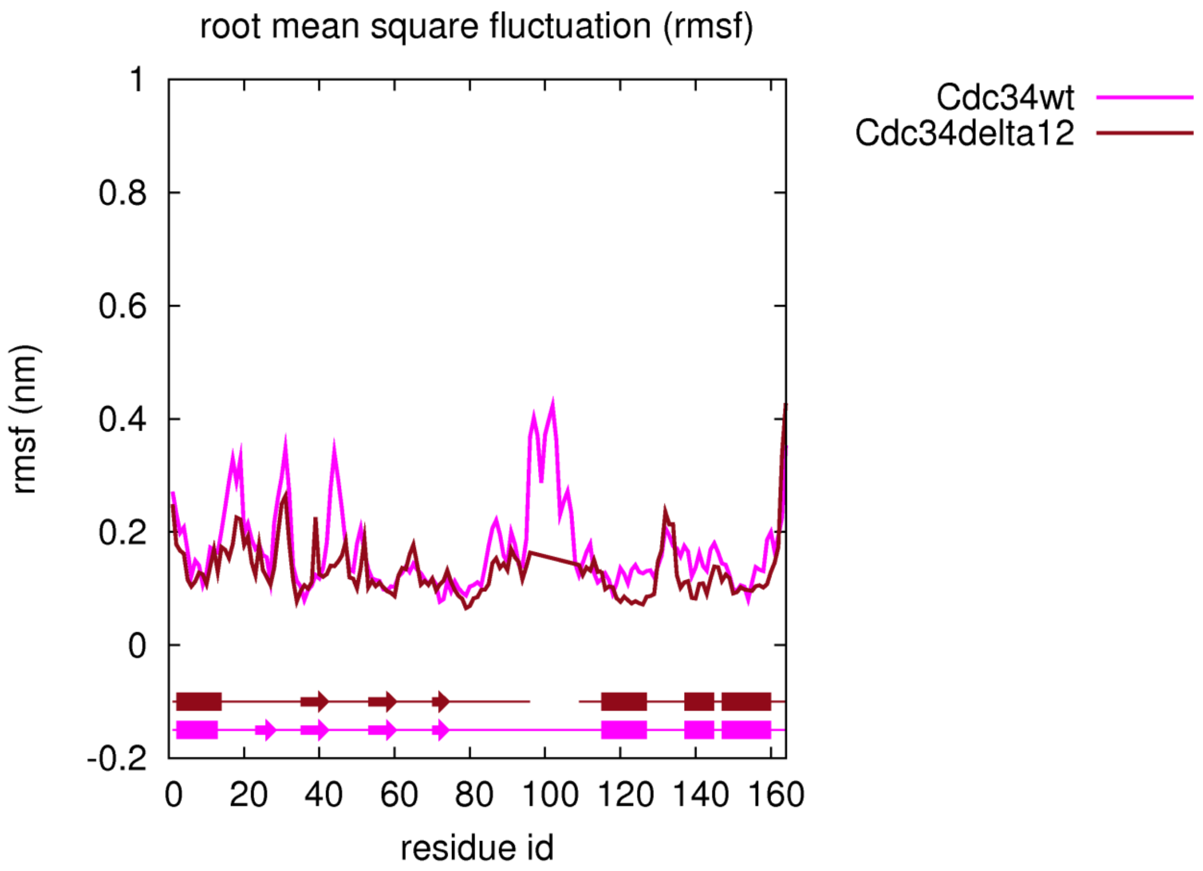

Figure 8. Model of Cdc34 both in closed (A) and open (B) conformations in complex with Uba1 E1 on the base of the known crystallographic structures of E2-E1 enzymes (PDB codes: 3CMM (Uba1) and 2PX9 (Ubc9 E2 in complex with SAE2 E1) and 2NVU (Ubc12 E2 in complex with Uba3 E1 and Nedd8).

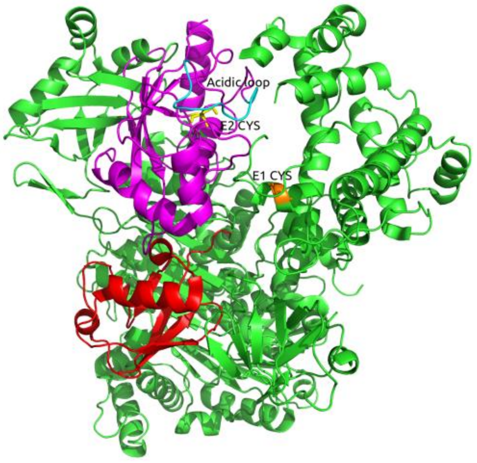

A

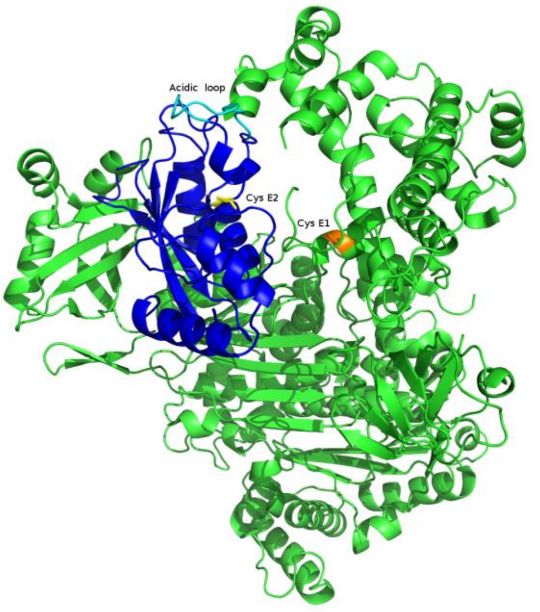

B

Figure 9. Mainchain root mean square deviation of single replicas of each simulated protein system.

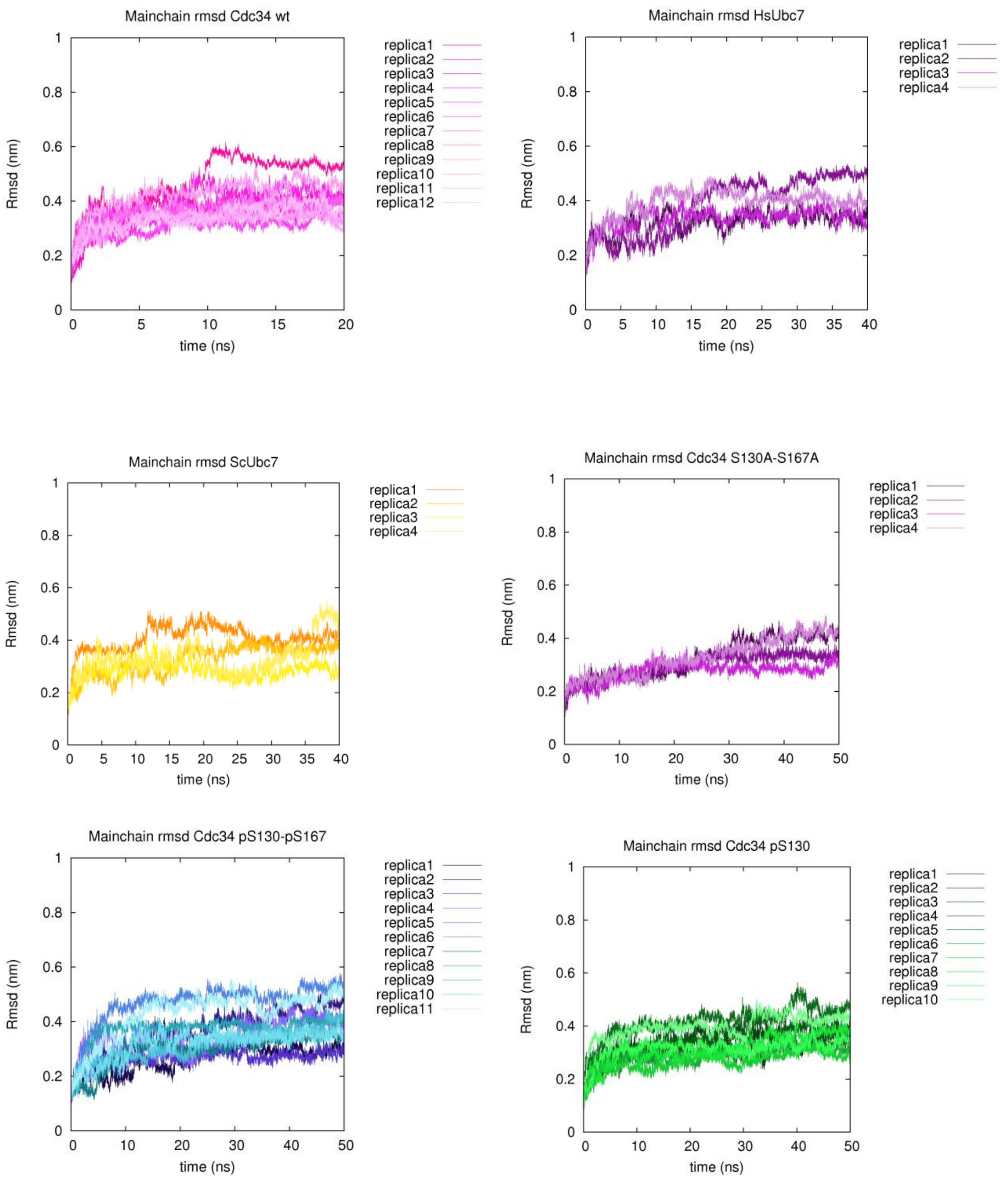

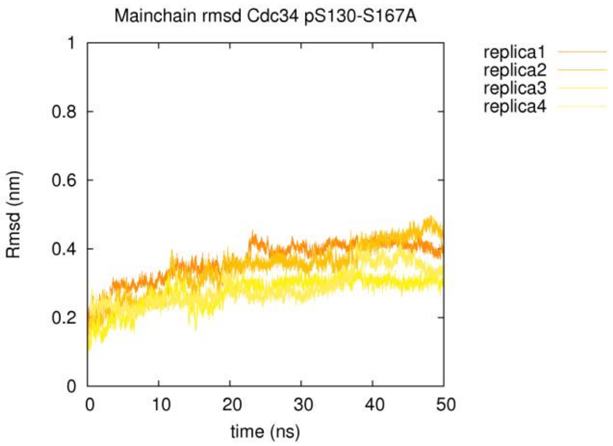
Mainchain rmsd Cdc34 pS130-S167A
Rmsd (nm)
0
0.2
0.4
0.6
0.8
1
0
10
20
30
40
50
time (ns)
replica1
replica2
replica3
replica4

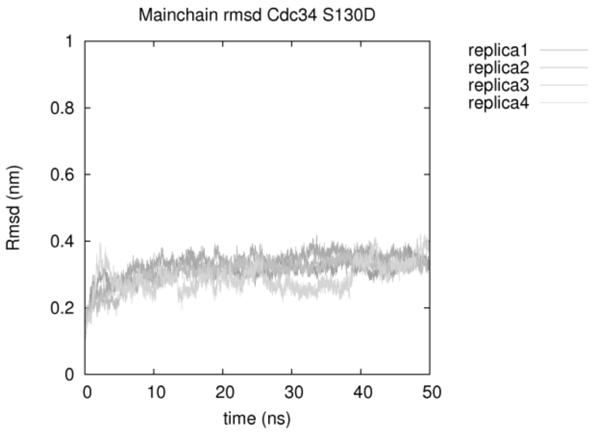
Mainchain rmsd Cdc34 S130D
Rmsd (nm)
0
0.2
0.4
0.6
0.8
1
0
10
20
30
40
50
time (ns)
replica1
replica2
replica3
replica4

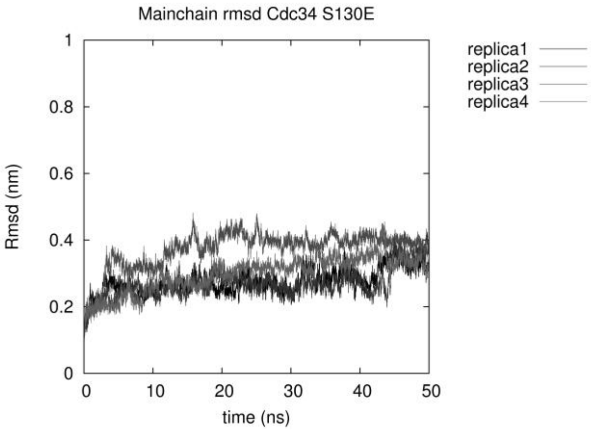
Mainchain rmsd Cdc34 S130E
Rmsd (nm)
0
0.2
0.4
0.6
0.8
1
0
10
20
30
40
50
time (ns)
replica1
replica2
replica3
replica4

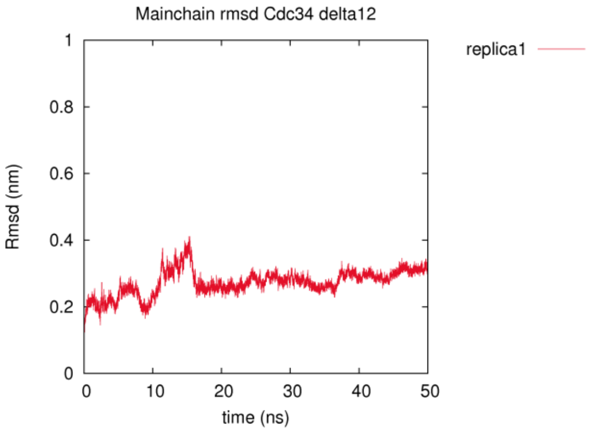
Mainchain rmsd Cdc34 delta12
Rmsd (nm)
0
0.2
0.4
0.6
0.8
1
0
10
20
30
40
50
time (ns)
replica1

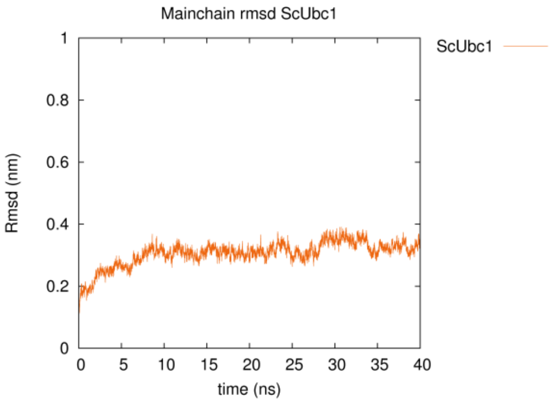
Mainchain rmsd ScUbc1
Rmsd (nm)
0
0.2
0.4
0.6
0.8
1
0
5
10
15
20
25
30
35
40
time (ns)
ScUbc1

Figure 10. Mainchain root mean square deviation of the structural elements of the common E2-fold (the mainchain atoms of the acidic loop are not included in the analysis) of the Cdc34 simulations.

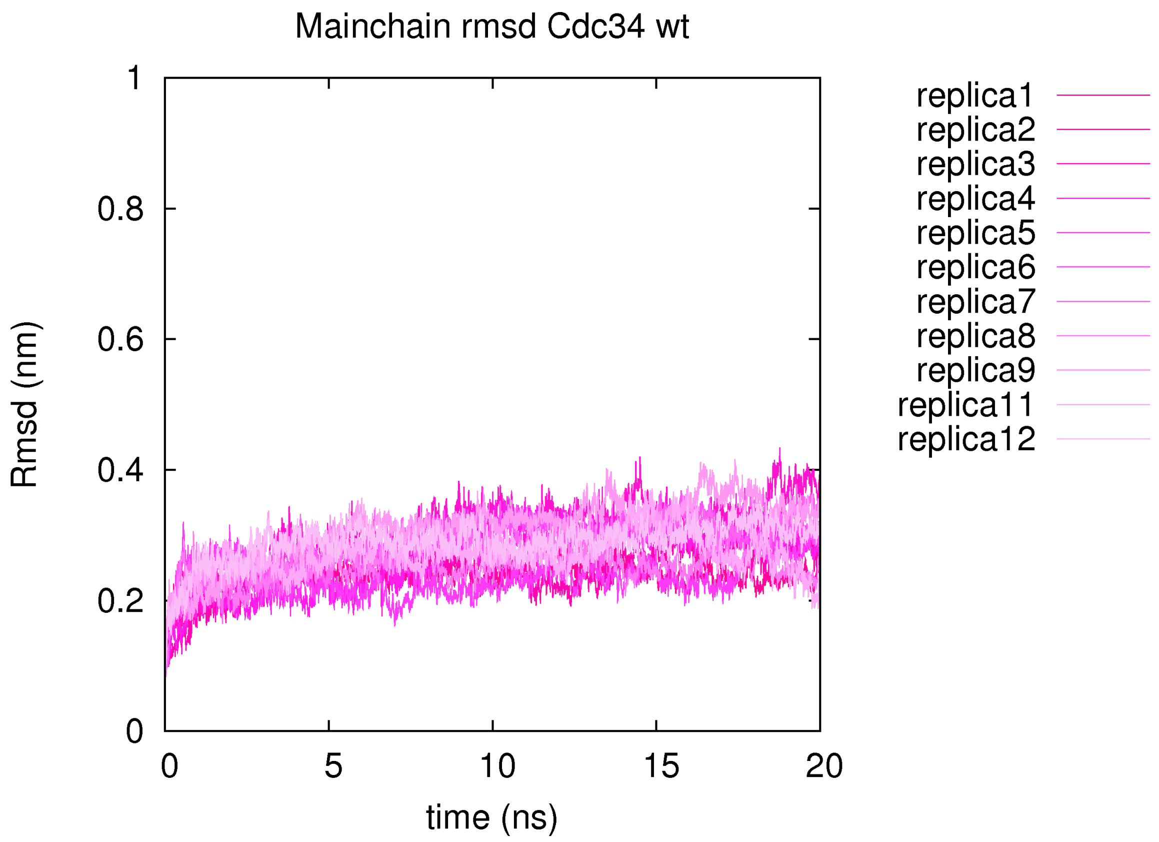

Figure 11. Cosine content along the first 20 principal components of single replicas and concatenated trajectories of different length for each simulated protein system.

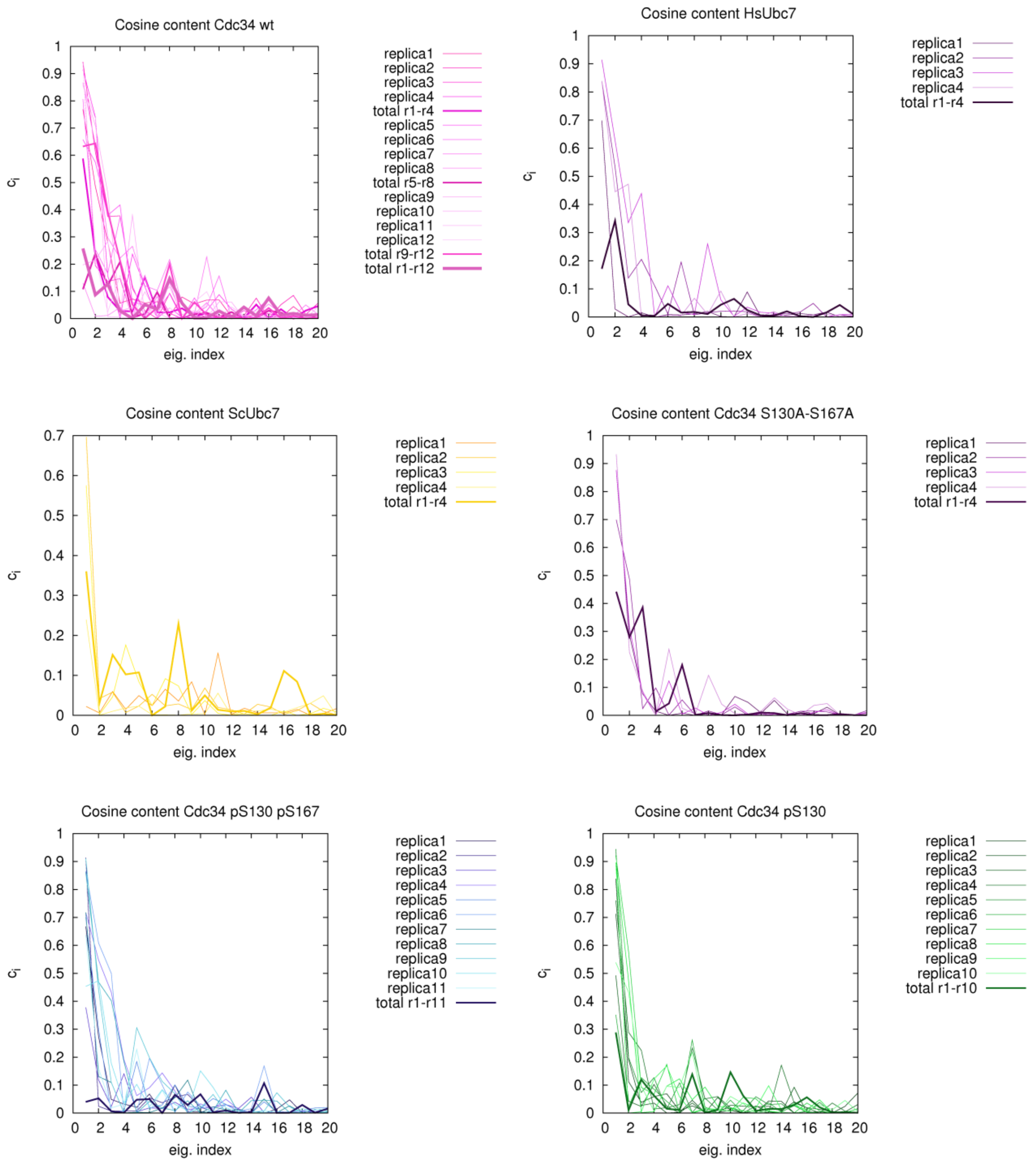

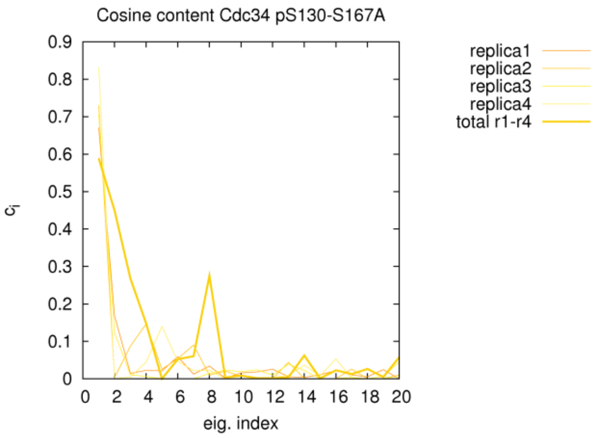
Cosine content Cdc34 pS130-S167A
c_i
eig. index
replica1
replica2
replica3
replica4
total r1-r4

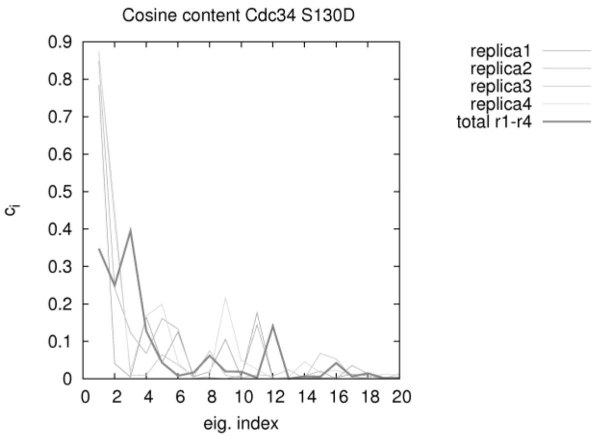
Cosine content Cdc34 S130D
c_i
eig. index
replica1
replica2
replica3
replica4
total r1-r4

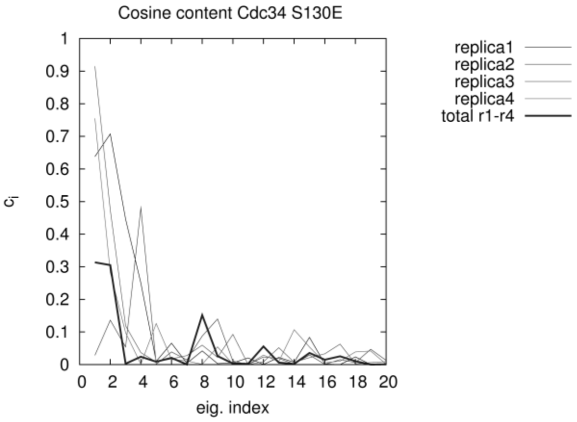
Cosine content Cdc34 S130E
c_i
eig. index
replica1
replica2
replica3
replica4
total r1-r4

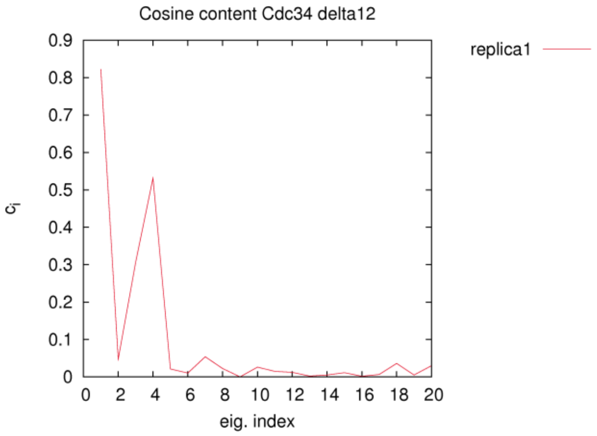
Cosine content Cdc34 delta12
c_i
eig. index
replica1
