# Supplementary material for: An Acidic Loop and Cognate Phosphorylation Sites Define a Molecular Switch That Modulates Ubiquitin Charging Activity in Cdc34-Like Enzymes
Source: PLoS Comput Biol. 2011 May 26;7(5):e1002056. doi: 10.1371/journal.pcbi.1002056 (PMC3102755; doi:10.1371/journal.pcbi.1002056)
Supplement: Text S2 — Homology modeling of Cdc34UBC to generate starting structures for molecular dynamics simulations and molecular dynamics simulations setup and analysis. (PDF) [file pcbi.1002056.s002.pdf]

Text S2. Homology modeling of Cdc34^{UBC} to generate starting structures for molecular dynamics simulations and molecular dynamics simulations setup and analysis.

Homology modelling of Cdc34^{UBC} to generate starting structures for simulations.

Three different models have been generated with Modeller version 9.6 for *S. cerevisiae* Cdc34 UBC domain (7-170 residues, Cdc34^{UBC}) using as templates the known X-ray structures of human [PDB code 2CYX], yeast [PDB code 2UCZ] and *C .elegans* [PDB code 1 PZV] Ubc7 enzymes, sharing with yeast Cdc34 more than 40% of sequence identity. The models quality was evaluated using Procheck, AIDE and Verify3D. Multiple sequence alignments between sequences of Cdc34 UBC domain and of its homologs with known three-dimensional (3D) structure were performed with ClustalW and T-coffee. The multiple alignments were compared and modified by hand according to information on functional and conserved residues, as wells an on secondary structures in order to get the optimal alignment for homology modeling.

Molecular dynamics simulations setup

Initially the system was relaxed by molecular mechanics (steepest descent, 5000 steps). The optimization step was followed by 50 ps of solvent equilibration at 300 K (time step 1 fs), while restraining the protein atomic positions using an harmonical potential. During equilibration the coupling constant to the external bath was set to 1 fs. The system was slowly driven to the target temperature (300 K) and pressure (1 bar) through a series of 50 ps thermalization and pressurization simulations.

In the MD simulations, all the protein atoms were at a distance equal or greater than 0.6 nm from the box edges. The ionization state of residues was set to be consistent with neutral pH and the tautomeric form of histidine residues was derived using GROMACS tools and confirmed by visual inspection and Propka server.

Productive MD simulations were performed in the isothermal-isobaric ensemble at 300 K, using an external bath with a coupling constant of 0.1 ps. Pressure was kept constant (1 bar) by modifying the box dimensions and the time-constant for pressure coupling was set to 1 ps. The LINCS algorithm was used to constrain heavy atom bonds, allowing the use of a 2 fs time-step. Electrostatic interactions were calculated using the Particle-mesh Ewald (PME) summation scheme. Van der Waals and Coulomb interactions were truncated at 1.0 nm. The non-bonded pair list was updated every 10 steps and conformations were stored every 4 ps.

Cluster analysis and Principal Component analysis of Cdc34^{UBC} simulations

The main-chain rmsd matrices have been computed on the concatenated macro-trajectories. The rmsd matrices have then been processed using both the Linkage and Gromos algorithms implemented in the Gromacs package to extract clusters of similar conformations. Different rmsd cutoff were tested for cluster analysis and they were selected according to the average rmsd values derived by the rmsd matrices. In

particular, for Cdc34^{UBC} simulations, the cutoffs which better resemble the similarity between conformations in the trajectories were 0.25 and 0.35 nm for Linkage and Gromos algorithms, respectively. Only clusters which collected at least 10% of the total structures were considered in order to filter out from the concatenated trajectories those structures which are not frequently sampled during the simulations. The Gromos and Linkage algorithm gave similar results, differing only in the number of structures in the less populated clusters. Therefore, only the results of Gromos clustering are discussed. The average structure of each cluster is defined as the protein structure with the lowest average distance (rmsd) to all other structures belonging to the same cluster.

To achieve a representation of the FEL, we define the probability of finding the system in a particular state characterized by a value q_α of the variables of interest (*reaction coordinates*) as proportional to ($e\text{-}G^{\alpha/kT}$) where Gα is the free energy of the state. A FEL estimation can be obtained from: $G\alpha = -kT\,ln[P(q_\alpha)/P_{max}(q)]$ where k is the Boltzmann constant, T is the temperature of simulation, $P(q_\alpha)$ is an estimate of the probability density function obtained by histograms of MD data and $P_{max}(q)$ is the probability of the most probable state. Considering two different reaction coordinates q_i and q_j, the two-dimensional FEL was obtained from the joint probability distributions $P(q_i, q_j)$.

The cosine content (c_i) of the principal components (PCs) of the covariance matrix is an absolute measure that can be extracted from covariance analysis. It has been demonstrated that insufficient sampling lead to high c_i values, representative of random motions, and which cannot be interpreted in terms of characteristic features of the conformational landscape. c_i was calculated on the first 20 PCs of each single replica and concatenated trajectories of different duration (Figure 11S, Supporting Information). The cosine content of the concatenated trajectories was closed to zero, confirming that the simulations collected for each system were sufficient to obtain a reliable conformational sampling. The analysis of the sampling convergence has been further assessed by computing the root mean square inner product (rmsip) as a measure of similarity between subspaces, which has been computed on the first 10 eigenvectors.

The conformational sub-states distribution in the proximity of the native-state of Cdc34^{UBC} has been evaluated by free energy landscape (FEL) (2D) representation using as 2D coordinates the first two principal components (PCs) derived by principal components analysis (Figure 2S) and then verified including also the third PC and by structural cluster analysis (*data not shown*). We identified several densely populated regions in the conformational sub-space, which can be considered as basins in the conformational landscape since they suggest the tendency of the system to adopt structures associated to those basins. The structural cluster analysis and the free energy landscape (FEL) representation using as reaction coordinates the first and the second principal components (PCs) indicate that Cdc34^{UBC} exists in several sub-states in solution (Figure 2S, 4A). The comparison between the main basins on the FEL and the most

populated structural clusters of Cdc34^{UBC} allowed to identify five main structural *ensembles* (Figure 2S), capturing the most relevant conformational and dynamical features of Cdc34^{UBC}.
